# New Dibenzo-*α*-pyrone Derivatives with *α*-Glucosidase Inhibitory Activities from the Marine-Derived Fungus *Alternaria alternata*

**DOI:** 10.3390/md20120778

**Published:** 2022-12-14

**Authors:** Jinxin Zhang, Baodan Zhang, Lei Cai, Ling Liu

**Affiliations:** 1 State Key Laboratory of Mycology, Institute of Microbiology, Chinese Academy of Sciences, Beijing 100101, China; 2 University of Chinese Academy of Sciences, Beijing 100049, China

**Keywords:** marine-derived fungus, *Alternaria alternata*, OSMAC, dibenzo-*α*-pyrone, biological activities, antioxidant, *α*-glucosidase inhibition, molecular docking

## Abstract

Three new dibenzo-*α*-pyrone derivatives, alternolides A–C (**1**–**3**), and seven known congeners (**4**–**10**) were isolated from the marine-derived fungus of *Alternaria alternata* LW37 assisted by the one strain-many compounds (OSMAC) strategy. The structures of **1**–**3** were established by extensive spectroscopic analyses, and their absolute configurations were determined by modified Snatzke′s method and electronic circular dichroism (ECD) calculations. Compounds **6** and **7** showed good 1,1-diphenyl-2-picrylhydrazyl (DPPH) antioxidant scavenging activities with IC_50_ values of 83.94 ± 4.14 and 23.60 ± 1.23 µM, respectively. Additionally, **2**, **3** and **7** exhibited inhibitory effects against *α*-glucosidase with IC_50_ values of 725.85 ± 4.75, 451.25 ± 6.95 and 6.27 ± 0.68 µM, respectively. The enzyme kinetics study indicated **2** and **3** were mixed-type inhibitors of *α*-glucosidase with *K*_i_ values of 347.0 and 108.5 µM, respectively. Furthermore, the interactions of **2**, **3** and **7** with *α*-glucosidase were investigated by molecular docking.

## 1. Introduction

Dibenzo-*α*-pyrones are polyketides containing a 6*H*-benzo[*c*]-chromen-6-one tricyclic skeleton and are abundant in fungi, but mainly from *Alternaria*, bacteria, lichens and high plants [1,2,3]. Up to now, more than 61 dibenzo-*α*-pyrones have been reported [2], and some of them exhibited a wide spectrum of biological properties such as brine shrimp lethality [4] and cytotoxic [5,6], mycotoxic [7], larvicidal [8] and antimicrobial activities [1,3,9], which have attracted much more attention from pharmaceutical scientists [2,10,11]. Total syntheses of several bioactive dibenzo-*α*-pyrones such as alternariol, alternariol 9-methyl ether and dehydroaltenuene A have been accomplished [12,13]. Dibenzo-*α*-pyrones are key intermediates in the synthesis of cannabinoids [14,15], as well as agonists of progesterone and glucocorticoid receptors [16,17]. Biosynthetically, fungal dibenzo-*α*-pyrones could be derived from acetyl-CoA and malonyl-CoA under the catalysis of polyketide synthase (PKS), followed by addition, enolization, and oxidation reactions [2,18]. *α*-Glucosidase causes the release of *α*-glucopyranose by hydrolyzing the terminal non-reducing residues of various carbohydrate substrates [19,20]. Inhibiting the activity of *α*-glucosidase can help treat carbohydrate-dependent diseases such as diabetes and obesity [21,22]. Microorganisms are considered to be rich sources of *α*-glucosidase inhibitors [23]. However, due to the microbial resource scarcity in the general environment, pharmacists have focused on special habitat microorganisms, hoping to find new *α*-glucosidase inhibitors [24].

More than 70% of the Earth’s surface is covered by oceans, and numerous marine-derived fungi have been isolated and identified in oceanic sediments, sponges, algae, etc. [25]. Marine-derived fungi survive extreme conditions such as absence of light, low levels of oxygen and intensely high pressures, which may result in unique biological metabolic pathways, and were considered to be a rich source of structurally diverse and biologically active metabolites for drug discovery [26,27,28]. *Alternaria* species have a widespread distribution in nature, acting as plant (include marine algae) pathogens, endophytes and saprophytes [29,30,31,32,33]. *A. alternata* is an extremely common and cosmopolitan species occur in many types of plant, soil and marine environments [34,35]. The fungal genus *Alternaria* can produce diverse secondary metabolites including dibenzo-*α*-pyrones [2], terpenoids [36] and polyketides [37,38], which show a broad range of biological activities such as antibacterial [2], anti-inflammatory [36], acetylcholinesterase inhibitory [37] and cytotoxic activities [38]. The secondary metabolites from marine-derived *Alternaria* sp. are also endowed with unique structures and varieties of bioactivities, such as the anti-inflammatory agent tricycloalternarene A possessing the unique fusion of an oxaspiro[5.5]nonane and a cyclohexenone ring [39], the cytotoxic agent altertoxin VII featured by a perylenequinone skeleton [40], and the antibacterial agent alternaramide with cyclic pentadepsipeptide skeleton [41].

**Figure 1:**
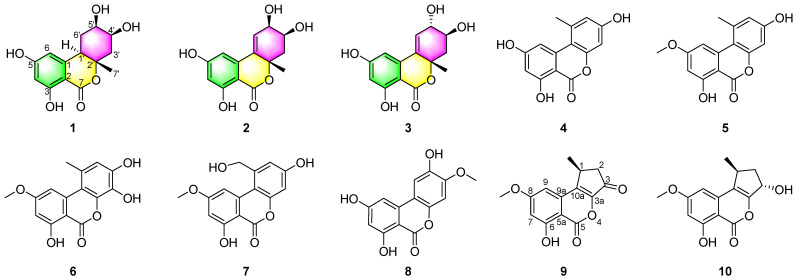
Structures of compounds **1**–**10**.

As part of our ongoing search for bioactive metabolites from the marine-derived fungi [42,43], *A. alternata* LW37, a fungus isolated from a deep-sea sediment sample collected at a depth of 2623 m in the Southwest Indian Ridge in November 2014, was screened for chemical investigation. The fungus *A. alternata* LW37 was then cultured in six different media (Table S1) guided by the OSMAC strategy [44]. Analysis of HPLC fingerprints (Figure S1) showed that the metabolic profile of this fungus on rice is more productive than those on other media. The fungus was cultured on rice for large-scale fermentation. Chemical investigation of the EtOAc extract of the fungus *A. alternata* LW37 led to the isolation of three new dibenzo-*α*-pyrones derivatives, alternolides A–C (**1**–**3**), and seven known compounds **4**−**10** (Figure 1). The isolated compounds were evaluated for their cytotoxic, antioxidant and *α*-glucosidase inhibitory activities. Details of the isolation, structure elucidation and biological evaluation of these metabolites are reported herein.

## 2. Results

**Figure 2:**
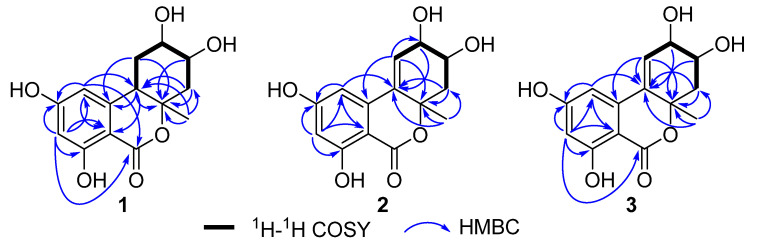
Key ^1^H-^1^H COSY and HMBC correlations of **1**–**3**.

Alternolide A (**1**) was isolated as a yellow oil, and its molecular formula was established as C_14_H_16_O_6_ based on the high-resolution electrospray ionization mass spectrometry (HRESIMS) data at *m*/*z* 281.1026 [M + H]^+^ (calcd for C_14_H_17_O_6_ 281.1020), indicating 7 degrees of unsaturation. The infrared (IR) absorptions at 3375, 1722, 1629 and 1463 cm^−1^ suggested the presence of hydroxy, lactone and phenyl, respectively. The ^1^H (nuclear magnetic resonance) NMR spectrum (Table 1) of **1** displayed signals of two aromatic protons *δ*_H_ 6.27 (s), 6.21 (s), two oxymethine protons *δ*_H_ 4.11 (m), 3.86 (m), one methine proton *δ*_H_ 3.14 (d, *J* = 12.4 Hz), two pairs of methylene protons *δ*_H_ 1.71 (q, *J* = 12.4 Hz), 2.06 (dd, *J* = 12.4, 3.1 Hz), 2.23 (dd, *J* = 12.4, 3.3 Hz), 2.23 (dd, *J* = 12.4, 3.3 Hz), and one methyl *δ*_H_ 1.36 (s). The ^13^C NMR data (Table 1) together with heteronuclear single quantum correlations (HSQC) (Figure S6) data revealed 14 carbon resonances, including one ester carbonyl carbon (*δ*_C_ 170.6), six aromatic carbons (*δ*_C_ 166.6, 165.6, 145.0, 105.1, 101.8 and 101.6), two oxymethine carbons (*δ*_C_ 72.2 and 70.0), two methylene carbons (*δ*_C_ 43.4 and 28.5), one oxygenated tertiary carbon (*δ*_C_ 84.7), one methine carbon (*δ*_C_ 43.5), and one methyl carbon (*δ*_C_ 20.9). These data accounted for all ^1^H and ^13^C NMR resonances except for four unobserved exchangeable protons and suggested that **1** was a tricyclic compound with one phenyl subunit. The ^1^H-^1^H correlation spectroscopy (COSY) spectrum (Figure S7) of **1** showing the correlations of H_2_-3′/H-4′/H-5′/H_2_-6′/H-1′ (Figure 2), together with heteronuclear multiple bond correlations (HMBC) (Figure 2) from H-1′ to C-2′, C-3′ and C-7′, from H_2_-3′ to C-1′, C-2′ and C-7′, from H-4′ and H-6′ to C-2′, and from H_3_-7′ to C-1′, C-2′ and C-3′ established the cyclohexane moiety. Other HMBC correlations from aromatic proton H-4 to C-2, C-3, C-5, C-6 and the ester carbonyl carbon C-7 and from H-6 to C-2, C-4, C-5 and C-7 indicated the presence of a 1,2,3,5-tetrasubstituted benzene ring with the ester carbonyl carbon C-7 located at C-2. In addition, key HMBC correlations from H-6 to C-1′ and from H-1′ to C-1, C-2 and C-6 led to the connection of the tetrasubstituted benzene ring to the cyclohexane moiety via the C-1–C-1′ single bond. The four hydroxyl groups were located at C-3, C-5, C-4′ and C-5′, respectively, which was supported by the chemical shift values for C-3 (*δ*_C_ 165.6), C-5 (*δ*_C_ 166.6), C-4′ (*δ*_C_ 70.0) and C-5′ (*δ*_C_ 72.2). Furthermore, considering one remaining degree of unsaturation and the ^13^C NMR chemical shifts of C-7 (*δ*_C_ 170.6) and C-2′ (*δ*_C_ 84.7), both carbons were connected to the same oxygen atom to form lactone moiety, completing the 6*H*-benzo[*c*]chromen-6-one core skeleton. Thus, the planar structure of **1** was determined as depicted (Figure 1).

**Figure 3:**
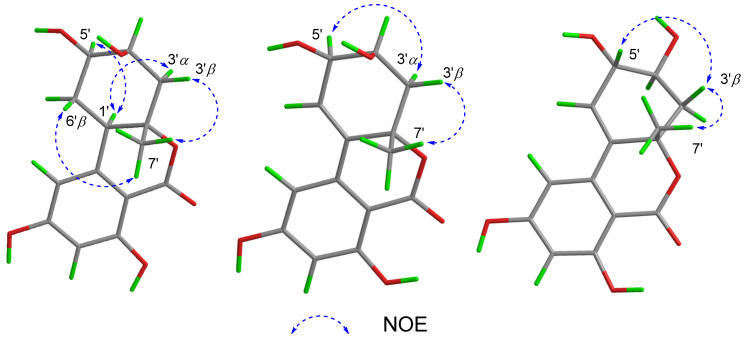
Key NOESY correlations of **1**–**3**.

The relative configuration of **1** was determined by analysis of the ^1^H-^1^H coupling constants and nuclear Overhauser effect spectroscopy (NOESY) data (Figure 3). The large coupling constants observed for H-6′*β*/H-1′ (*J* = 12.4 Hz) and H-6′*β*/H-5′ (*J* = 12.4 Hz) revealed their *trans*-diaxial orientations. The small vicinal coupling constants observed for H-4′/H-3′*α* (*J* = 3.1 Hz) and H-4′/H-3′*β* (*J* = 3.3 Hz) suggested the equatorial orientation of H-4′. The NOESY correlations of H-1′ with both H-3′*α* and H-5′ indicated that these protons are on the same side of the cyclohexane ring as axial orientations. Other NOESY correlations of H-6′*β* with H_3_-7′ and of H_3_-7′ with H-3′*β* defined these protons on the other side of the cyclohexane ring, indicating the *trans*-fusion of the cyclohexane and lactone rings.

**Figure 4:**
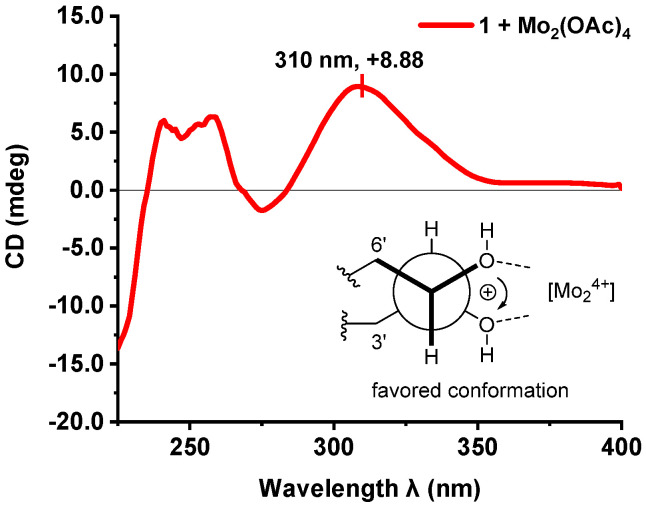
ECD spectrum of **1** in DMSO containing [Mo_2_(OAc)_4_] with the inherent ECD spectrum subtracted.

**Figure 5:**
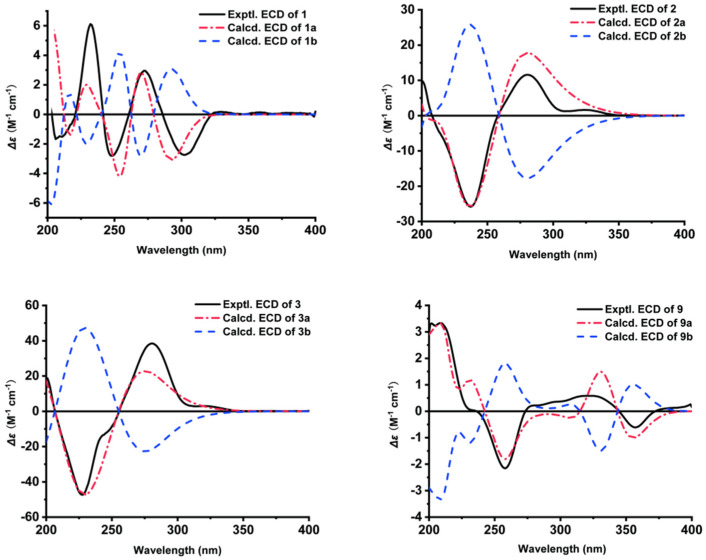
The calculated and experimental ECD spectra of **1**–**3** and **9**.

The absolute configuration of the 4′,5′-diol moiety in **1** was established by the Mo_2_(OAc)_4_-induced ECD experiment developed by Santzke [45,46]. As shown in Figure 4, the positive Cotton effect at 310 nm observed in the Mo_2_(OAc)_4_-induced ECD spectrum of **1** indicated the 4′*S* and 5′*R* configurations. Therefore, the absolute configuration of **1** was assigned as 1′*R*,2′*S*,4′*S*,5′*R*. This inference was further supported by comparison of the experimental and calculated ECD spectra (Figure 5). The simulated ECD spectra of (1′*R*,2′*S*,4′*S*,5′*R*)*-***1** (**1a**) and (1′*S*,2′*R*,4′*R*,5′*S)-***1** (**1b**) were generated by the time-dependent density functional theory (TDDFT), and the experimental ECD spectra of **1** were in good agreement with the calculated ECD spectrum for **1a**. Thus, the structure of **1** was then demonstrated as depicted.

Alternolide B (**2**) was also obtained as a yellow oil. The molecular formula was determined as C_14_H_14_O_6_ (eight degrees of unsaturation) by HRESIMS (*m*/*z* 279.0872 [M + H]^+^), which is two mass units less than that of **1**. The ^1^H and ^13^C NMR data (Table 1) of **2** were similar to those of **1**, with the exception of the absence of one methine (*δ*_H/C_ 3.14/43.5, C-1′) and one methylene (*δ*_H/C_ 1.71; 2.23/28.5, C-6′) signal and the presence of the additionally trisubstituted double bond signals (*δ*_C_ 135.2, C-1′; *δ*_H/C_ 6.16/129.7, C-6′). This was further supported by the HMBC correlations from H-3′, H-5′ and H_3_-7′ to C-1′, and from H-6′ to C-1, C-2′ and C-5′ (Figure 2), as well as the ^1^H-^1^H COSY correlations (Figure 2) of H_2_-3′/H-4′/H-5′/H-6′. Consequently, the gross structure of **2** was established (Figure 1).

The relative configuration of **2** was also determined by ^1^H-^1^H coupling constants (Table 1) and NOESY data (Figure 3). The small coupling constants observed for H-4′/H-3′*α* (*J* = 2.8 Hz) and H-4′/H-3′*β* (*J* = 6.3 Hz) revealed the pseudo-equatorial orientation of H-4′. The NOESY correlation (Figure 3) of H-5′ with H-3′*α* suggested that H-5′ and H-3′*α* are cofacial and pseudoaxial orientations, while NOESY correlation of H-7′ with H-3′*β* defined these protons as on the opposite face the cyclohexene ring. To establish the absolute configuration of **2**, the ECD spectrum of **2** was recorded in MeOH and compared with the calculated spectra of a pair of enantiomers, (1′*S*,4′*S*,5′*R*)-**2** (**2a**) and (1′*R*,4′*R*,5′*S*)-**2** (**2b**). The experimental ECD spectrum of **2** was consistent with the one calculated for **2a** (Figure 5), allowing the assignment of the absolute configuration of **2** as 1′*S*,4′*S*,5′*R*.

Alternolide C (**3**) was obtained as a yellow oil and its molecular formula was determined to be C_14_H_14_O_6_ (eight degrees of unsaturation) based on the HRESIMS ion peaks at *m*/*z* 279.0868 [M + H]^+^ (calcd for 279.0863), which were the same as those of **2**. Comparing the ^1^H and ^13^C NMR data (Table 1) with those of **2** revealed that **2** and **3** are almost the same, with slight differences in the chemical shifts of C-4′ and C-5′ (*δ*_H/C_ 4.12/68.2, C-4′ in **2** vs. *δ*_H/C_ 3.78/70.7, C-4′ in **3**; *δ*_H/C_ 4.37/68.4, C-5′ in **2** vs. *δ*_H/C_ 4.07/72.3, C-5′ in **3**), indicating the remarkable structural similarity between **2** and **3**. Detailed analysis of the ^1^H-^1^H COSY and HMBC correlations (Figure 2) revealed the same planar structure as that of **2**, suggesting their diastereomeric relationship. The relative configuration of **3** was also deduced from ^1^H-^1^H coupling constants (Table 1) and NOESY correlations (Figure 2). The large *trans*-diaxial-type *J* value of 9.4 Hz for H-3′*β* and H-4′ revealed their *trans*-diaxial orientations. The NOESY correlations (Figure 3) of H-3′*β* with H-5′ and H_3_-7′ indicated that these protons were on the same face of the cyclohexene ring, while the H-4′ was on the opposite face of the cyclohexene ring. Thus, the relative configuration was established.

The absolute configurations of C-1′, C-4′ and C-5′ in **3** were also deduced by comparison of the experimental spectrum of **3** with the calculated ECD spectra for a pair of enantiomers, (1′*S*,4′*S*,5′*S*)-**3** (**3a**) and (1′*R*,4′*R*,5′*R*)-**3** (**3b**). The calculated ECD spectrum of (1′*S*,4′*S*,5′*S*)-**3** (**3a**) showed good agreement with the experimental curve (Figure 5), which supported the absolute configuration as being 1′*S*,4′*S*,5′*S*. Thus, the completed structure of **3** was elucidated as depicted (Figure 1).

Compound **9** was identified as 1-deoxyrubralactone by comparison of ^1^H and ^13^C NMR spectroscopic data and optical rotation with those reported previously in the literature [47]. However, its absolute configuration had never been reported before. Through a comparison of the experimental spectrum of **9** with the calculated ECD spectra for the enantiomers (1*S*)-**9** (**9a**) and (1*R*)-**9** (**9b**), we observed that the calculated ECD spectrum of **9a** showed good agreement with the experimental one (Figure 5). Thus, the absolute configuration of **9** was determined as 1*S* (Figure 1).

The known compounds alternariol (**4**) [5], alternariol 5-*O*-methyl ether (**5**) [5], 3′-hydroxyalternariol 5-*O*-methyl ether (**6**) [5], alternariol 1′-hydroxy-9-methyl ether (**7**) [48], altenuisol (**8**) [49], and phialophoriol (**10**) [50] were determined by comparison of their spectroscopic data with those in the literature.

**Figure 6:**
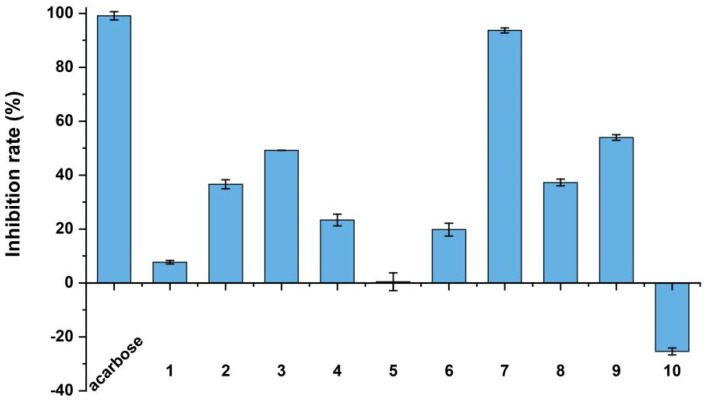
The *α*-glucosidase inhibitory activities of **1**–**10**. Acarbose was used as a positive control.

Compounds **1**–**3** were tested for their cytotoxic activities against B16 (mouse melanoma cells), MCF-7 (human breast carcinoma cells) and HepG2 (human hepatocellular carcinoma cells). However, these compounds did not show detectable inhibitory effects on the cell lines tested at 50 μM. Additionally, all of the isolated compounds were tested for their antioxidative activity against DPPH and *α*-glucosidase inhibitory activities. Compounds **6** and **7** showed good DPPH antioxidant scavenging activities with IC_50_ values of 83.94 ± 4.14 and 23.60 ± 1.23 µM, respectively, whereas the corresponding positive control, ascorbic acid, showed an IC_50_ value of 23.70 ± 1.03 µM. *α*-Glucosidase inhibition assay results showed that compounds **2**, **3**, **7**, **8** and **9** exerted *α*-glucosidase inhibitory activities with inhibition rates of 36.62%, 49.24%, 93.70%, 37.29% and 53.95%, respectively, at a concentration of 400 µM (Figure 6). Compounds **2** and **3** exhibited inhibition on *α*-glucosidase with IC_50_ values of 725.85 ± 4.75 and 451.25 ± 6.95 μM, respectively, while compound **7** showed significant inhibitory activity with an IC_50_ value of 6.27 ± 0.68 µM (the positive control, acarbose, showed an IC_50_ value of 1.59 ± 1.37 μM). Acarbose is one of the three *α*-glucosidase inhibitors in clinics for the treatment of diabetes.

**Figure 7:**
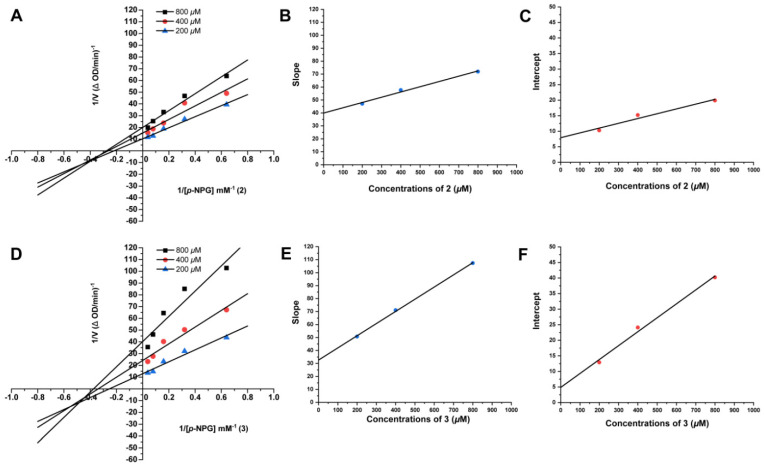
The Lineweaver–Burk and secondary plots of **2** (**A**–**C**) and **3** (**D**–**F**) for *α*-glucosidase inhibition.

In order to gain a better understanding of the *α*-glucosidase inhibition patterns of **2** and **3**, Lineweaver−Burk plots were applied. In the Lineweaver−Burk plots (Figure 7A,D), both *K*_m_ and *V*_max_ values of compounds **2** and **3** decreased with increasing concentration, and the lines of **2** and **3** intersected at the third quadrants. These results suggested that compounds **2** and **3** were mixed-type inhibitors against *α*-glucosidase, indicating that they were able to bind either the free *α*-glucosidase or the *α*-glucosidase–substrate complex. By the secondary plots (Figure 7B,C,E,F) of the slope and intercept versus concentrations, their *K*_is_ values (the inhibition constant of the enzyme−substrate complex) were calculated as 982.5 and 513.5 μM, respectively, and *K*_i_ values (the inhibition constant of the free enzyme) were 347.0 and 108.5 μM, respectively. The *K*_i_ values were smaller than their *K*_is_ values, indicating the priority in binding with the free enzyme.

**Figure 8:**
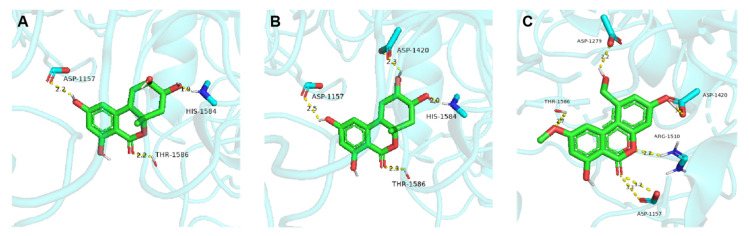
Molecular docking simulations of *α*-glucosidase with bioactive compounds **2** (**A**), **3** (**B**) and **7** (**C**).

To investigate the molecular interactions between compounds (**2**, **3**, and **7**) and *α*-glucosidase, a molecular docking study was performed using the program AutoDock Vina 1.1.2. The binding modes predicted for compounds **2**, **3**, and **7** are shown in Figure 8. Compound **2** formed three hydrogen bonds with the Asp1157, His1584 and Thr1586 residues, and **3** formed four hydrogen bonds with the Asp1157, Asp1420, His1584 and Thr1586 residues. Compound **7** formed six hydrogen bonds with Asp1157, Asp1279, Asp1420, Arg1510 and Thr1586 residues (Figure 8C). The docking results of **2** and **3** revealed that different relative configurations of 4′,5′-diol unit caused change in the binding mode. It can be argued that the 5′-OH with the absolute configuration *S*, forming a hydrogen bond with Asp1420, can enhance the *α*-glucosidase inhibition activity of this class of dibenzo-*α*-pyrones. This conclusion is consistent with the experimental results for enzyme activity.

## 3. Experimental Section

### 3.1. General Experimental Procedure

Optical rotations were measured with an Anton Paar MCP 200 Automatic Polarimeter (Anton Paar, Graz, Austria). The UV data were recorded on a Thermo Genesys-10S UV/Vis spectrophotometer (Thermo Fisher Scientific, Waltham, MA, USA). ECD spectra were recorded with a JASCO J-815 spectropolarimeter (JASCO, Tsukuba, Japan) by using CH_3_OH as the solvent. Infrared spectra were obtained on a Nicolet IS5 FT-IR spectrophotometer (Thermo Fisher Scientific, Waltham, MA, USA). ^1^H and ^13^C NMR spectroscopic data were acquired with a Bruker Avance-500 spectrometer (Bruker, Bremen, Germany) using the solvent signals as a reference (CD_3_OD: *δ*_H_ 3.31/*δ*_C_ 49.00). HRESIMS data were obtained using an Agilent Accurate-Mass-Q-TOF LC/MS 6520 instrument (Agilent Technologies, Santa Clara, CA, USA) equipped with an electrospray ionization (ESI) source. Semi-preparative HPLC separation was performed on an Agilent 1260 instrument equipped with a variable-wavelength UV detector (Agilent Technologies Inc., CA, USA) using a YMC-pack ODS-A (10 × 250 mm, 5 μm, 2 mL/min, YMC CO., LTD., Kyoto, Japan). Open column chromatography (CC) was performed on a Sephadex LH–20 (GE Healthcare, Uppsala, Sweden) and silica gel (200–300 mesh, Qingdao Marine Chemical Factory, Qingdao, China), respectively. *α*-Glucosidase (from *Saccharomyces cerevisiae*, 33 U/mg), *p*-nitrophenyl-*α*-D-glucopyranoside (*p*-NPG) and acarbose were purchased from Shanghai Yuanye Bio-Technology Co., Ltd. (Shanghai, China).

### 3.2. Strain and Fermentation

The fungal strain *A. alternata* LW37 was isolated from a deep-sea sediment sample collected at a depth of 2623 m in the Southwest Indian Ridge in November 2014. Phylogenetic analyses (Figure S2) based on *LSU*, *SSU*, ITS and *RPB2* sequences and morphological features (Figure S3) indicated that LW37 should be identified as the known species *A. alternata*, deposited in GenBank as accessions OP316895 (ITS), OP326732 (*LSU*), OP326733 (*SSU*) and OP326734 (*RPB2*), and in the culture collection at the Institute of Microbiology, Chinese Academy of Sciences, Beijing.

The strain was cultured on potato dextrose agar (PDA) plates at 25 °C for 5 d. Additionally, the plugs of agar, supporting mycelial growth, were cut from solid culture medium and transferred aseptically to 250 mL Erlenmeyer flasks, each containing 50 mL liquid medium (0.4% glucose, 1% malt extract and 0.4% yeast extract). Flask cultures were incubated at 28 °C on a rotary shaker at 170 rpm for 5 d to obtain the seed culture. Later, a large-scale fermentation of *A. alternata* LW37 was performed in solid rice medium using 500 mL × 40 conical flasks for 30 d at 28 °C, and each flask contained 100 g of rice, 110 mL water and 1 mL of the seed culture.

### 3.3. Extraction and Isolation

The fermented rice material was extracted repeatedly with EtOAc (3 × 5.0 L), and the organic solvent was evaporated to dryness to afford the crude extract (35.0 g). The extract was fractionated by silica gel CC using petroleum ether (PE)/EtOAc (8:1–1:2) gradient elution to give four fractions (Fr. 1−4). The fraction 2 (1.98 g, eluted with PE/EtOAc 1:1) was subjected to octadecylsilyl column chromatography (ODS CC) with MeOH-H_2_O gradient elution to yield seven subfractions (Fr. 2-1−2-7). The subfraction 2-7 (42.7 mg, eluted with 80% MeOH-H_2_O) was purified by semi-preparative RP-HPLC (85% MeOH-H_2_O for 30 min; 2.0 mL/min) to afford compounds **4** (6.1 mg, *t*_R_ 16.2 min), and **5** (3.9 mg, *t*_R_ 27.0 min). The subfraction 2-5 (57.1 mg, eluted with 50% MeOH-H_2_O) was purified by semi-preparative RP-HPLC (45% CH_3_CN-H_2_O for 45 min; 2.0 mL/min) to afford compounds **8** (2.2 mg, *t*_R_ 24.4 min), **10** (5.0 mg, *t*_R_ 29.7 min) and **9** (3.4 mg, *t*_R_ 39.5 min). The subfraction 2-5 (44.3 mg, eluted with 70% MeOH-H_2_O) was purified by semi-preparative RP-HPLC (65% CH_3_CN-H_2_O for 15 min; 2.0 mL/min) to afford compound **6** (7.8 mg, *t*_R_ 12.2 min). The subfraction 2-3 (29.7 mg, eluted with 30% MeOH-H_2_O) was purified by semi-preparative RP-HPLC (45% CH_3_CN-H_2_O for 15 min; 2.0 mL/min) to afford compound **7** (3.4 mg, *t*_R_ 16.5 min). The fraction 3 (0.57 g, eluted with PE/EtOAc 1:2) was subjected to octadecylsilyl column chromatography (ODS CC) with MeOH-H_2_O gradient elution to yield six subfractions (Fr. 3-1−3-6). The subfraction 3-5 (236.7 mg, eluted with PE/EtOAc 1:1) was purified by semi-preparative RP-HPLC (22% CH_3_CN-H_2_O for 60 min; 2.0 mL/min) to afford compounds **3** (7.2 mg, *t*_R_ 46.2 min), **1** (3.6 mg, *t*_R_ 48.5 min) and **2** (1.7 mg, *t*_R_ 53.2 min).

Alternolide A (**1**): yellow oil; [*α*${]}_{D}^{25}$ = −4.0 (*c* 0.1, CH_3_OH); UV (MeOH) *λ*_max_ (log *ε*) 210 (1.99), 226 (1.87), 271 (1.78), 305 (1.49) nm; IR (neat) *ν*_max_ 3375, 2949, 1722, 1629, 1463, 1361, 1266, 1170, 1077, 983, 849 cm^−1^; ECD (4.3 × 10^−3^ M) *λ*_max_ (Δ*ε*) 211 (−1.52), 232 (+6.12), 248 (−2.81), 272 (+2.95), 302 (−2.75); positive HRESIMS at *m*/*z* 281.1026 [M + H]^+^ (calcd for C_14_H_17_O_6_ *m*/*z* 281.1020).

Alternolide B (**2**): yellow oil; [*α*${]}_{D}^{25}$ = −1.0 (*c* 0.1, CH_3_OH); UV (MeOH) *λ*_max_ (log *ε*) 205 (1.87), 243 (2.15), 281 (1.82), 320 (1.55) nm; IR (neat) *ν*_max_ 3382, 2931, 1652, 1441, 1350, 1270, 1195, 1086, 978, 849 cm^−1^; ECD (1.8 × 10^−2^ M) *λ*_max_ (Δ*ε*) 237 (−25.81), 252 (−6.85), 280 (+11.58); positive HRESIMS at *m*/*z* 279.0872 [M + H]^+^ (calcd for C_14_H_15_O_6_ *m*/*z* 279.0863).

Alternolide C (**3**): yellow oil; [*α*${]}_{D}^{25}$ = +18.0 (*c* 0.1, CH_3_OH); UV (MeOH) *λ*_max_ (log *ε*) 210 (2.11), 242 (1.95), 280 (1.55), 320 (1.25) nm; IR (neat) *ν*_max_ 3401, 2927, 1654, 1467, 1347, 1271, 1174, 1068, 934, 851 cm^−1^; ECD (2.9×10^−2^ M) *λ*_max_ (Δ*ε*) 228 (−47.32), 240 (−16.84), 280 (+38.45); positive HRESIMS at *m*/*z* 279.0868 [M + H]^+^ (calcd for C_14_H_15_O_6_ *m*/*z* 279.0863).

Alternariol (**4**): [*α*${]}_{D}^{25}$ = 0.0 (*c* 0.1, CH_3_OH).

Alternariol 5-*O*-methyl ether (**5**): [*α*${]}_{D}^{25}$ = 0.0 (*c* 0.1, CH_3_OH).

3′-Hydroxyalternariol 5-*O*-methyl ether (**6**): [*α*${]}_{D}^{25}$ = 0.0 (*c* 0.1, CH_3_OH).

Alternariol 1′-hydroxy-9-methyl ether (**7**): [*α*${]}_{D}^{25}$ = 0.0 (*c* 0.1, CH_3_OH).

Altenuisol (**8**): [*α*${]}_{D}^{25}$ = 0.0 (*c* 0.1, CH_3_OH).

1-Deoxyrubralactone (**9**): [*α*${]}_{D}^{25}$ = −2.0 (*c* 0.1, CH_3_OH); ECD (1.2×10^−3^ M) *λ*_max_ (Δ*ε*) 209 (+3.33), 258 (−2.16), 357 (−0.61).

Phialophoriol (**10**): [*α*${]}_{D}^{25}$ = +95.0 (*c* 0.1, CH_3_OH).

### 3.4. Absolute Configuration of the 4′,5′-Diol Moiety in 1

Snatzke’s method was used to determine the absolute configuration of the 4′,5′-diol moiety in **1**. Dissolve 0.3 mg of **1** and 0.36 mg of Mo_2_(OAc)_4_ in dry DMSO to produce a solution at a compound concentration of 0.8 mg/mL. After mixing, the first ECD was recorded immediately, and the additional induced ECD spectra were recorded every 5 min until reaching the stationary state. The inherent ECD spectrum was subtracted. The absolute configuration of the 4′,5′-diol for compound was demonstrated by the sign at around 310 nm in the observed ECD spectrum.

### 3.5. ECD Calculation

Conformational analysis of compounds **1**–**3** within an energy window of 3.0 kcal/mol was performed by using the OPLS3 molecular mechanics force field. The conformers were then further optimized with the software package Gaussian 09 [51] at the B3LYP/6-311G(d,p) level, and the harmonic vibrational frequencies were also calculated to confirm their stability. Then, the 60 lowest electronic transitions for the obtained conformers in vacuum were calculated using time-dependent density functional theory (TD-DFT) methods at the B3LYP/6-311G(d,p) level. ECD spectra of the conformers were simulated using a Gaussian function. The overall theoretical ECD spectra were obtained according to the Boltzmann weighting of each conformer.

### 3.6. Bioassays for Cytotoxic Activity

The cytotoxicity evaluations were performed according to the previously described protocol [42].

### 3.7. Antioxidant Assay

The DPPH antioxidant scavenging assay was performed according to the previously reported method [52]. Briefly, 50 µL of DPPH (0.34 mmol/L in EtOH) and 50 µL of a series of solutions (12.5, 25, 50, 100, and 200 μM) of the test compounds **1**−**10** were mixed in the wells of 96-well plates. Each mixture was incubated at 37 °C for 30 min in a dark environment. The absorbance was read at 517 nm using a microplate reader, employing distilled water as a blank for baseline correction. All experiments were performed in triplicate, and ascorbic acid was used as a positive control.

### 3.8. Bioassays for α-Glucosidase Inhibition Assay

The *α*-glucosidase inhibitory activity assay was measured as described in previous reports [43,53]. Briefly, 50 μL of 0.5 U/mL *α*-glucosidase and 25 μL of a series of solutions (0.1, 0.2, 0.4, 0.8 and 1.6 mM) of the test compounds **1**−**10** were added into 96-well plates. After incubation at 37 °C for 10 min, 25 μL of 25 mM *p*-NPG was added and further incubated at 37 °C for 10 min. The absorbances were determined at 405 nm on an automatic microplate reader, and acarbose was used as a positive control.

### 3.9. Enzyme Kinetics of α-Glucosidase Inhibition Assay

The inhibition types of compounds **2** and **3** on *α*-glucosidase were determined by Lineweaver−Burk plots according to a previous report [30]. The *α*-glucosidase inhibition kinetics were determined with selected concentrations of *p-*NPG (1.5625, 3.125, 6.25, 12.5 and 25 mM) under different concentrations of **2** and **3** (200, 400 and 800 μM) by keeping the enzyme concentration at 0.5 U/mL. The inhibition constant was determined by the second plots of the apparent *K*_m_/*V*_m_ or 1/*V*_m_ versus the concentration of the inhibitor.

### 3.10. Molecular Docking Assay

The molecular docking method was used to predict the possible binding sites of **2**, **3** and **7** with *α*-glucosidase [43]. The crystallographic structure of *α*-glucosidase from yeast (PDB ID: 3TOP) was obtained from the Protein Data Bank. Then, Chemdraw (20.0) and Chem3D (20.0) were used to obtain the chemical and MM2 energy-minimized 3D structures of compounds **2**, **3** and **7**. AutoDock Vina (1.1.2) was used to prepare the ligand and receptor and subsequent docking. Finally, pymol (2.4.0) was applied to visualize the interaction process for receptor and ligand.

## 4. Conclusions

In conclusion, three new dibenzo-*α*-pyrone derivatives, alternolides A–C (**1**–**3**), along with seven known compounds (**4**−**10**) were isolated from the crude extract of the marine-derived fungus *A. alternata* LW37 guided by OSMAC strategy. The structures of **1**–**3** were elucidated on the basis of spectroscopic data, modified Snatzke′s method and ECD calculations. Furthermore, we first reported the absolute configuration of 1-deoxyrubralactone (**9**). As for the bioactivities, the new compounds alternolides B and C were tested as mixed-type inhibitors against *α*-glucosidase with IC_50_ values of 725.85 ± 4.75 and 451.25 ± 6.95 μM, respectively. Unprecedentedly, we perceived that alternariol 1′-hydroxy-9-methyl ether (**7**) has promising *α*-glucosidase inhibition activity with an IC_50_ value of 6.27 ± 0.68 µM. Meanwhile, the molecular docking assay was used to determine the binding models of **2**, **3** and **7** with *α*-glucosidase. Based on the differences between the absolute configurations, experimental results of enzyme activity and molecular docking results of **2** and **3**, we speculated that the absolute configuration of 5′-OH had an effect on the *α*-glucosidase inhibitory activity of this kind of dibenzo-*α*-pyrone. This study not only provided a deeper insight into the chemical diversities and bioactivities of dibenzo-*α*-pyrones, but also demonstrated that marine-derived fungi represent promising producers of natural products with bioactivities for use in drug discovery and development.

## Figures and Tables

**Table 1:** ^1^H NMR and ^13^C NMR data (500 and 125 MHz) for **1**–**3** in CD_3_OD.

Position	1		2		3	
	*δ*_H_ (*J* in Hz)	*δ*_C_, mult.	*δ*_H_ (*J* in Hz)	*δ*_C_, mult.	*δ*_H_ (*J* in Hz)	*δ*_C_, mult.
1		145.0, C		140.7, C		141.1, C
2		101.6, C		100.6, C		100.6, C
3		165.6, C		165.2, C		165.2, C
4	6.21 (s)	101.8, CH	6.28 (d, 2.2)	103.6, CH	6.30 (d, 2.2)	103.5, CH
5		166.6, C		167.1, C		166.7, C
6	6.27 (s)	105.1, CH	6.51 (d, 2.2)	104.5, CH	6.52 (d, 2.2)	104.5, CH
7		170.6, C		170.4, C		170.5, C
1′	3.14 (d, 12.4)	43.5, CH		135.2, C		135.0, C
2′		84.7, C		82.4, C		82.3, C
3′*α*	2.06 (dd, 12.4, 3.1)	43.4, CH_2_	2.21 (dd, 14.0, 2.8)	40.8, CH_2_	2.40 (dd, 14.4, 3.9)	40.9, CH_2_
3′*β*	2.23 (dd, 12.4, 3.3)		2.38 (dd, 14.0, 6.3)		1.97 (dd, 14.4, 9.4)	
4′	4.11 (m)	70.0, CH	4.12 (m)	68.2, CH	3.78 (ddd, 9.4, 5.9, 3.9)	70.7, CH
5′	3.86 (m)	72.2, CH	4.37 (t, 3.3)	68.4, CH	4.07 (dd, 5.9, 2.8)	72.3, CH
6′*α*	2.23 (dd, 12.4, 3.3)	28.5, CH_2_	6.16 (d, 3.3)	129.7, CH	6.16 (d, 2.8)	131.0, CH
6′*β*	1.71 (q, 12.4)					
7′	1.36 (s)	20.9, CH_3_	1.61 (s)	27.9, CH_3_	1.50 (s)	28.0, CH_3_

## Data Availability

Data are contained within the article or Supplementary Material.

## Supplementary Materials

The following are available online at https://www.mdpi.com/article/10.3390/md20120778/s1. Table S1 and Figure S1: Details of OSMAC; Figures S2 and S3: Identification of LW37; Figures S4–S24: 1D and 2D NMR spectra and HRESIMS spectra of compounds **1**–**3**; Figures S25–S27: Infrared spectra of compounds **1**–**3**; Figures S28–S31: ECD spectra of compounds **1**–**3** and **9**; Figures S32–S35: ECD conformers of compounds **1**–**3** and **9**.
