# Supplementary material for: New Dibenzo-α-pyrone Derivatives with α-Glucosidase Inhibitory Activities from the Marine-Derived Fungus Alternaria alternata
Source: Mar Drugs. 2022 Dec 14;20(12):778. doi: 10.3390/md20120778 (PMC9785194; doi:10.3390/md20120778)
Supplement: Supplementary file 1 [file marinedrugs-20-00778-s001.zip › marinedrugs-2073667-supplementary.pdf]

Supplemental material

New dibenzo-α-pyrone derivatives with α-glucosidase inhibitory activities from the marine-derived fungus *Alternaria alternata*

Jinxin Zhang [1,2], Baodan Zhang [1,2], Lei Cai [1,2] and Ling Liu [1,2,*]

1 State Key Laboratory of Mycology, Institute of Microbiology, Chinese Academy of Sciences, Beijing 100101, China

2 University of Chinese Academy of Sciences, Beijing 100049, China

* Correspondence: liul@im.ac.cn; Tel.: +86-10-64807043

Table of Contents

Table S1. Component for the culture media S2
Figure S1. HPLC-UV analysis of metabolic profiles cultured in six media at 280 nm S2
Figure S2. Maximum likelihood tree of *Alternaria* sect. *Alternaria* based on sequences of *LSU*, *SSU*, ITS and *RPB2* S3
Figure S3. The colony and spore morphology of strain *A. alternata* LW37 S4
Figure S4. ^{1}H NMR spectrum of alternolide A (**1**; 500 MHz, CD_3OD) S5
Figure S5. ^{13}C NMR spectrum of alternolide A (**1**; 125 MHz, CD_3OD) S5
Figure S6. HSQC spectrum of alternolide A (**1**; 500 MHz, CD_3OD) S6
Figure S7. ^{1}H-^{1}H COSY spectrum of alternolide A (**1**; 500 MHz, CD_3OD) S6
Figure S8. HMBC spectrum of alternolide A (**1**; 500 MHz, CD_3OD) S7
Figure S9. NOESY spectrum of alternolide A (**1**; 500 MHz, CD_3OD) S7
Figure S10. ^{1}H NMR spectrum of alternolide B (**2**; 500 MHz, CD_3OD) S8
Figure S11. ^{13}C NMR spectrum of alternolide B (**2**; 125 MHz, CD_3OD) S8
Figure S12. HSQC spectrum of alternolide B (**2**; 500 MHz, CD_3OD) S9
Figure S13. ^{1}H-^{1}H COSY spectrum of alternolide B (**2**; 500 MHz, CD_3OD) S9
Figure S14. HMBC spectrum of alternolide B (**2**; 500 MHz, CD_3OD) S10
Figure S15. NOESY spectrum of alternolide B (**2**; 500 MHz, CD_3OD) S10
Figure S16. ^{1}H NMR spectrum of alternolide C (**3**; 500 MHz, CD_3OD) S11
Figure S17. ^{13}C NMR spectrum of alternolide C (**3**; 125 MHz, CD_3OD) S11
Figure S18. HSQC spectrum of alternolide C (**3**; 500 MHz, CD_3OD) S12
Figure S19. ^{1}H-^{1}H COSY spectrum of alternolide C (**3**; 500 MHz, CD_3OD) S12
Figure S20. HMBC spectrum of alternolide C (**3**; 500 MHz, CD_3OD) S13
Figure S21. NOESY spectrum of alternolide C (**3**; 500 MHz, CD_3OD) S13
Figure S22. HRESIMS spectrum of alternolide A (**1**) S14
Figure S23. HRESIMS spectrum of alternolide B (**2**) S14
Figure S24. HRESIMS spectrum of alternolide C (**3**) S14
Figure S25. Infrared spectrum of alternolide A (**1**) S15
Figure S26. Infrared spectrum of alternolide B (**2**) S15
Figure S27. Infrared spectrum of alternolide C (**3**) S15
Figure S28. ECD spectrum of alternolide A (**1**) S16
Figure S29. ECD spectrum of alternolide B (**2**) S16
Figure S30. ECD spectrum of alternolide C (**3**) S17
Figure S31. ECD spectrum of 1-deoxyrubralactone (**9**) S17
Figure S32. ECD conformers of alternolide A (**1**) S18
Figure S33. ECD conformers of alternolide B (**2**) S20
Figure S34. ECD conformers of alternolide C (**3**) S22
Figure S35. ECD conformers of 1-deoxyrubralactone (**9**) S25

Table S1 Component for the culture media

Media	Phase	Component
PDB	liquid	20% potato, 2% glucose
YES	liquid	15% sucrose, 2% yeast extract
SWS	liquid	1% starch, 0.1% peptone
YMG	liquid	0.4% glucose, 0.4% yeast extract, 1% maltose
GPY	liquid	1% glucose, 0.5% peptone, 0.1% yeast extract, 0.1% $CaCO_3$
Rice	solid	rice 100 g, distilled H_2O 110 mL

The liquid media incubated at 28°C for 7 days under on an orbital shaker at 200 rpm; The solid media incubated at 25°C under static condition for 30 days.

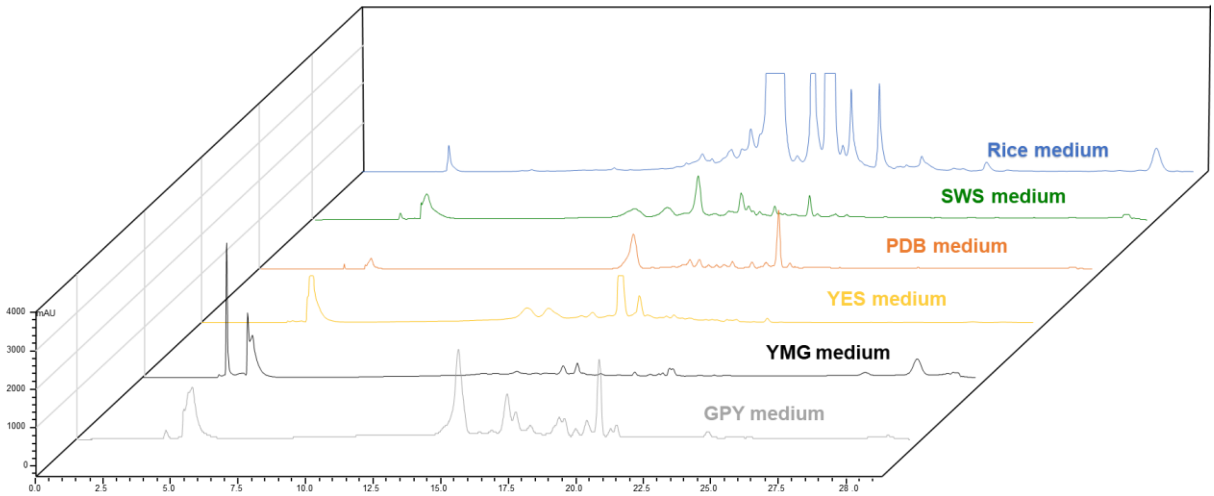

Figure S1. HPLC-UV analysis of metabolic profiles cultured in six media at 280 nm

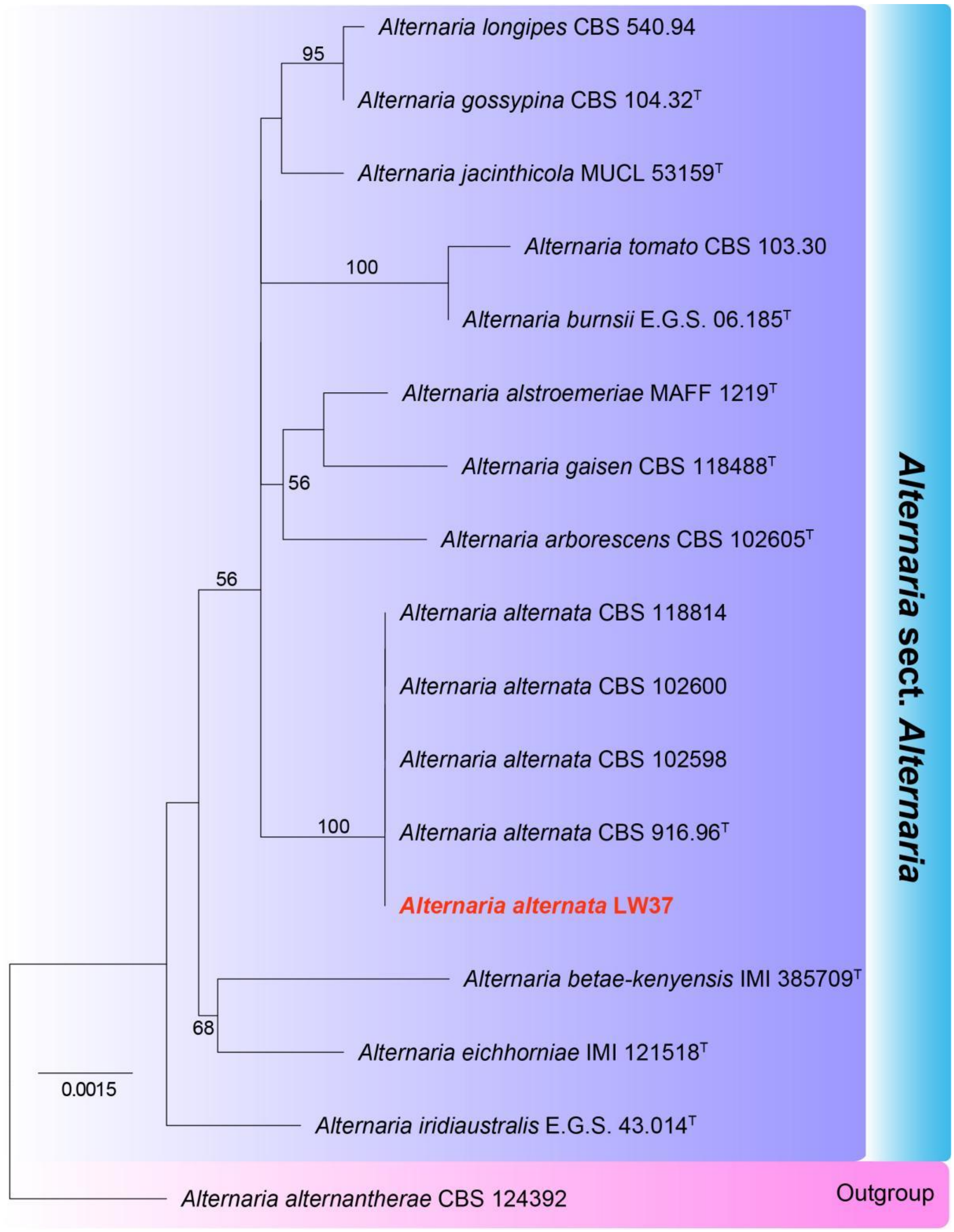

Figure S2. Maximum likelihood tree of *Alternaria* sect. *Alternaria* based on sequences of *LSU*, *SSU*, ITS and *RPB2*. Bootstrap support values of maximum likelihood (MLBP) above 50% are shown at the nodes. The tree was rooted to *Alternaria alternantherae* CBS 124392. Type cultures are indicated with a letter "T" after the accession number. The strain LW37 is printed in red and bold font.

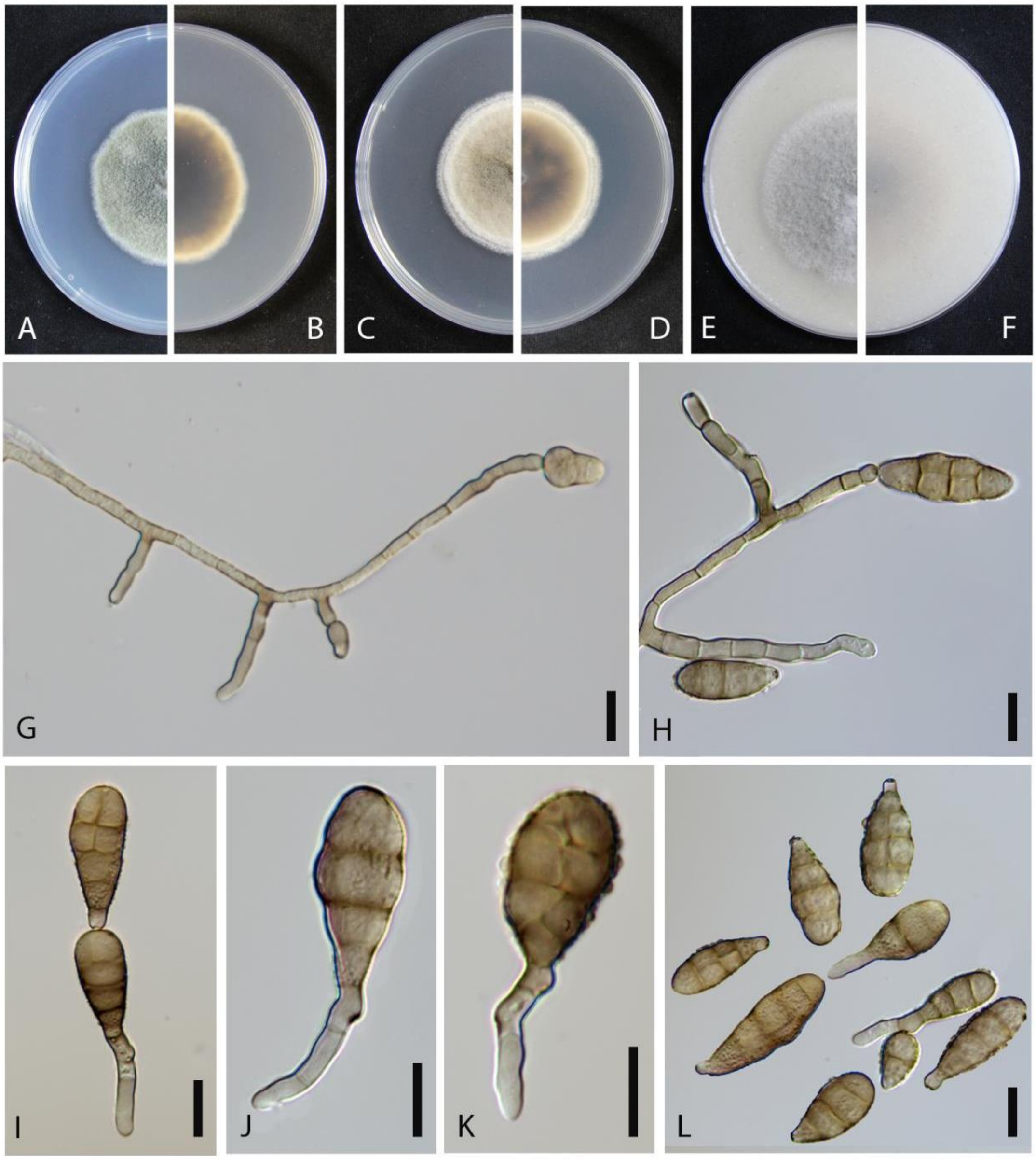

Figure S3. The colony and spore morphology of strain *Alternaria alternata* LW37. A, Surface and reverse of colony on PDA, MEA, OA and CYA. B, Sporulation on MEA. C-F, Conidiophores and conidiogenous cells. G, Conidia. Scale bars: C-G = 10 μm.

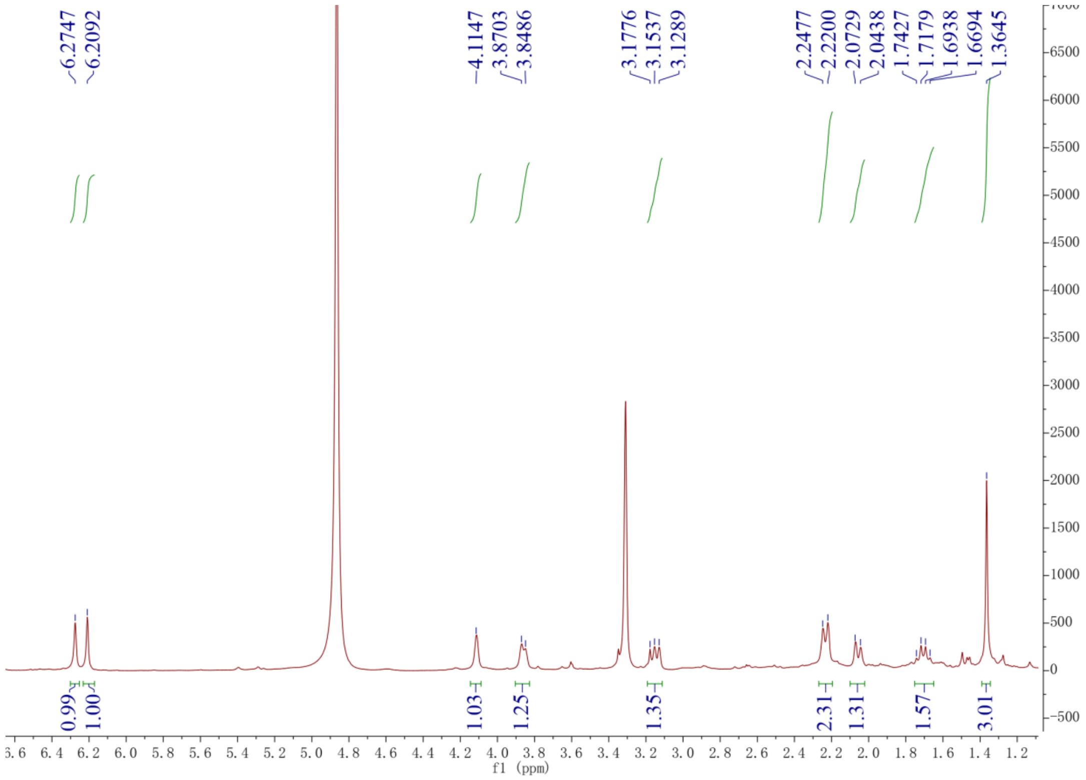

Figure S4. ^{1}H NMR spectrum of alternolide A (**1**; 500 MHz, CD_3OD)

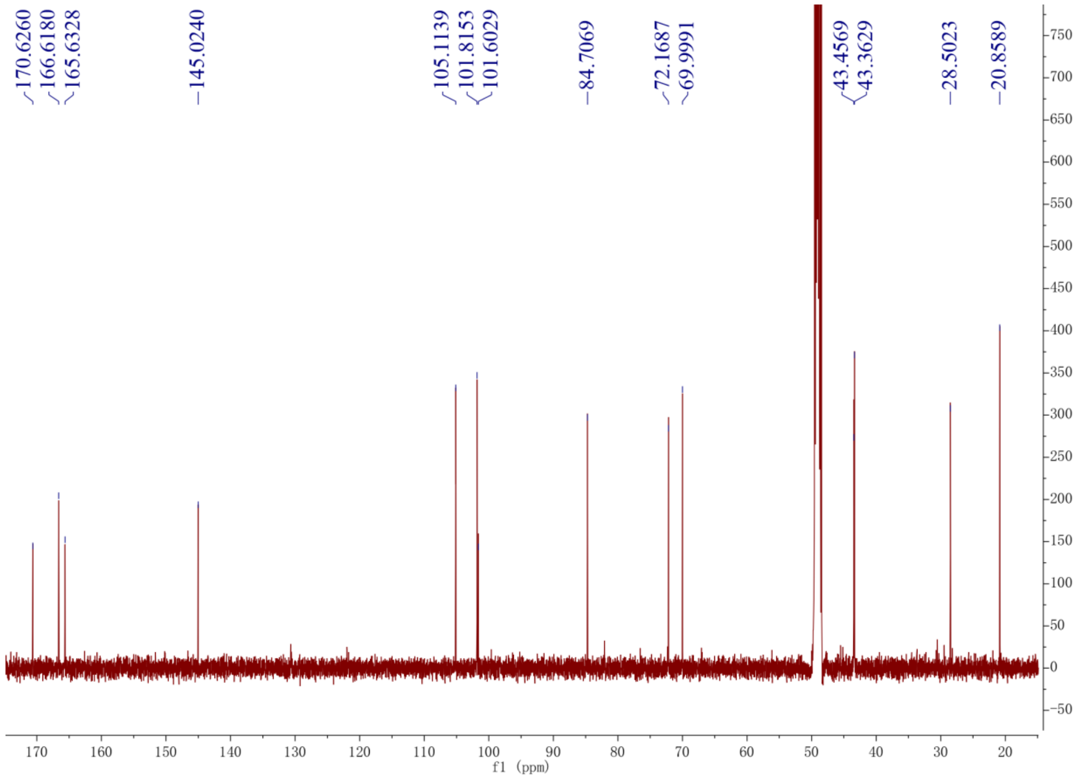

Figure S5. ^{13}C NMR spectrum of alternolide A (**1**; 125 MHz, CD_3OD)

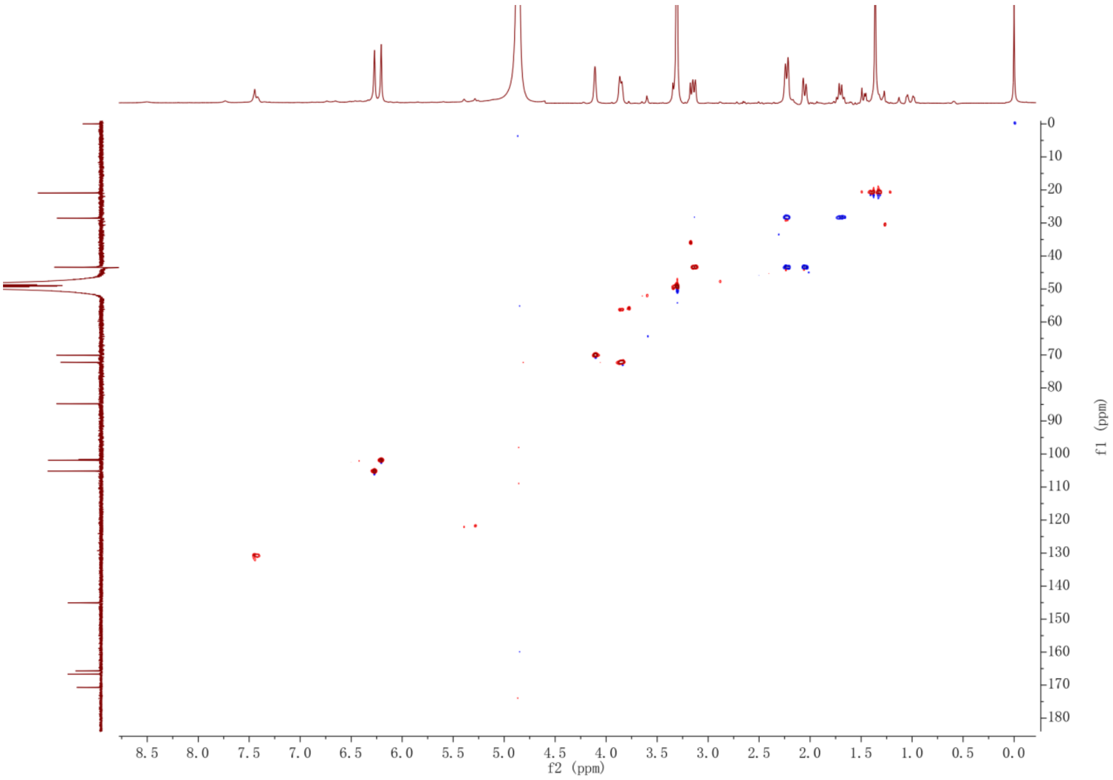

Figure S6. HSQC spectrum of alternolide A (**1**; 500 MHz, CD_3OD)

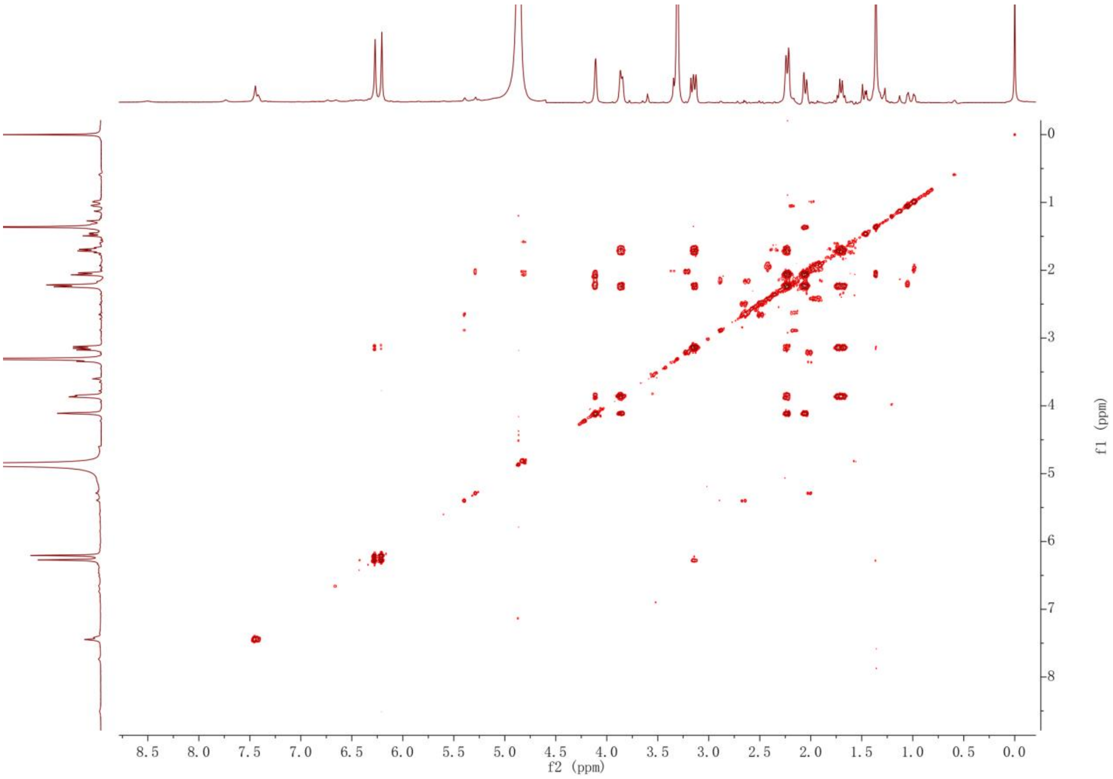

Figure S7. ^1H-^1H COSY spectrum of alternolide A (**1**; 500 MHz, CD_3OD)

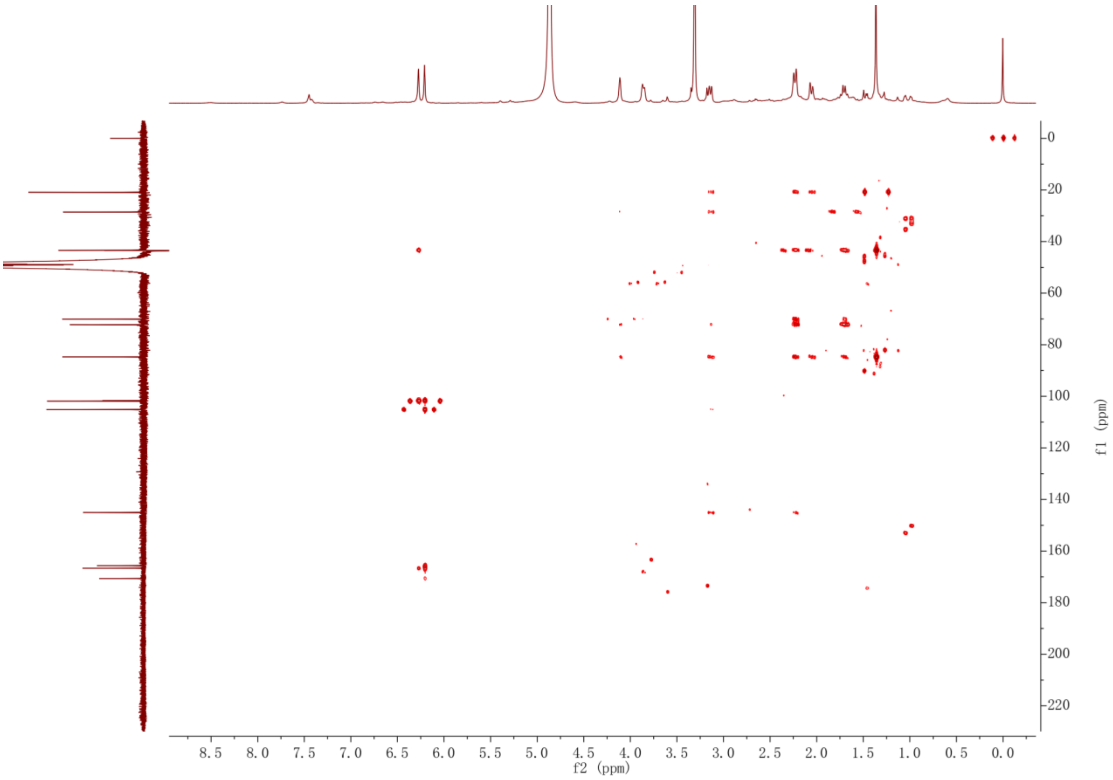

Figure S8. HMBC spectrum of alternolide A (**1**; 500 MHz, CD_3OD)

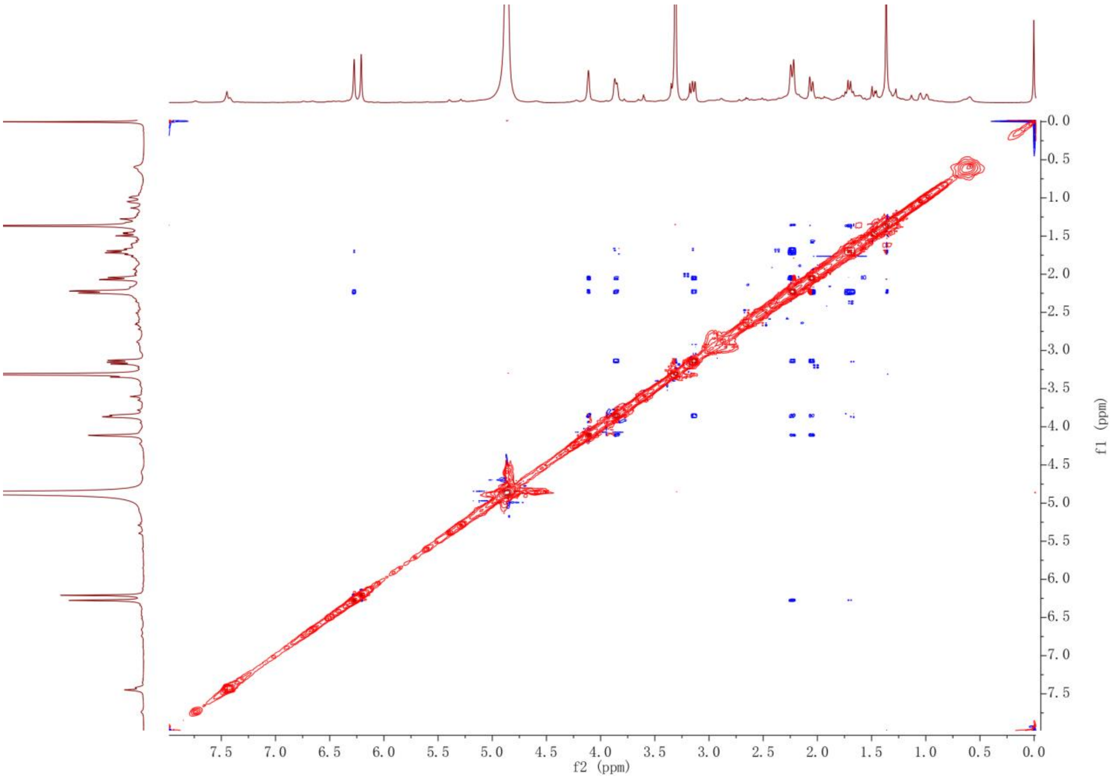

Figure S9. NOESY spectrum of alternolide A (**1**; 500 MHz, CD_3OD)

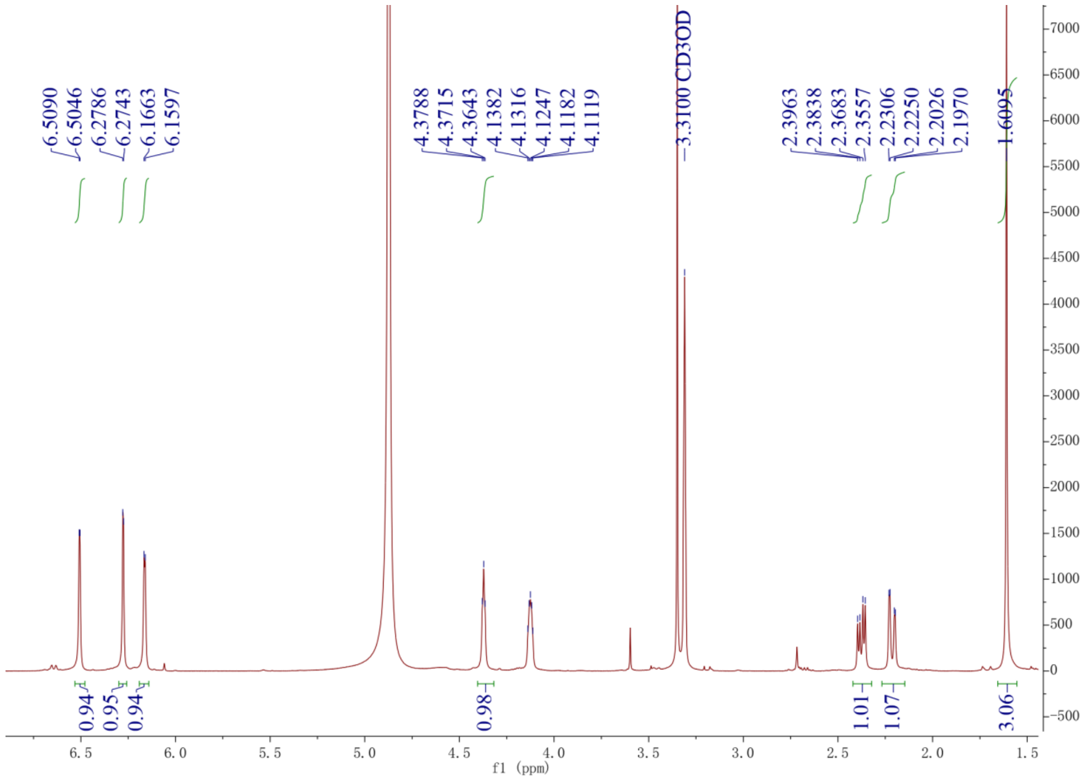

Figure S10. ^{1}H NMR spectrum of alternolide B (**2**; 500 MHz, CD_3OD)

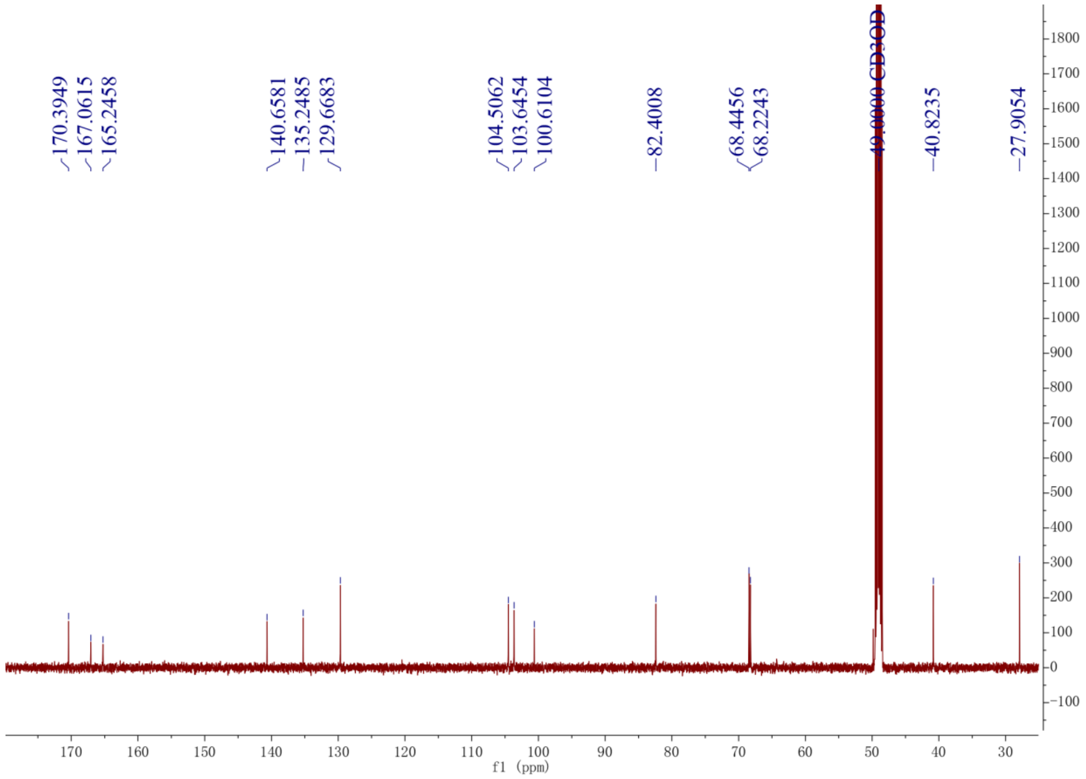

Figure S11. ^{13}C NMR spectrum of alternolide B (**2**; 125 MHz, CD_3OD)

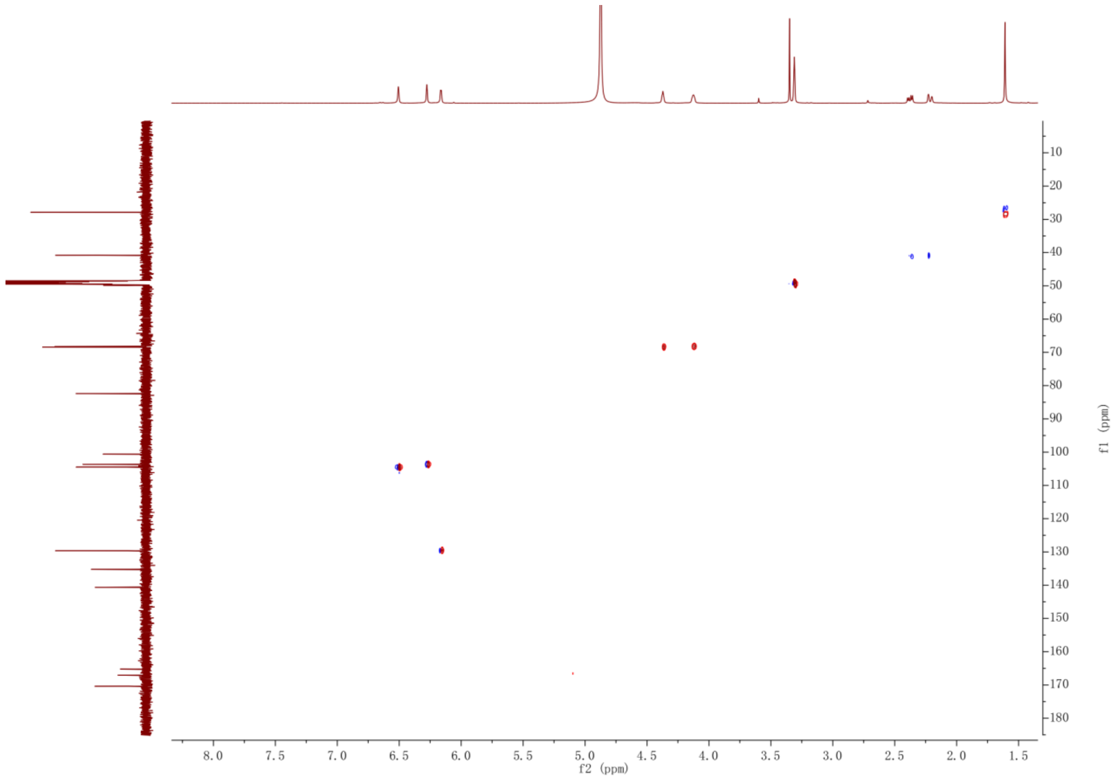

Figure S12. HSQC spectrum of alternolide B (**2**; 500 MHz, CD_3OD)

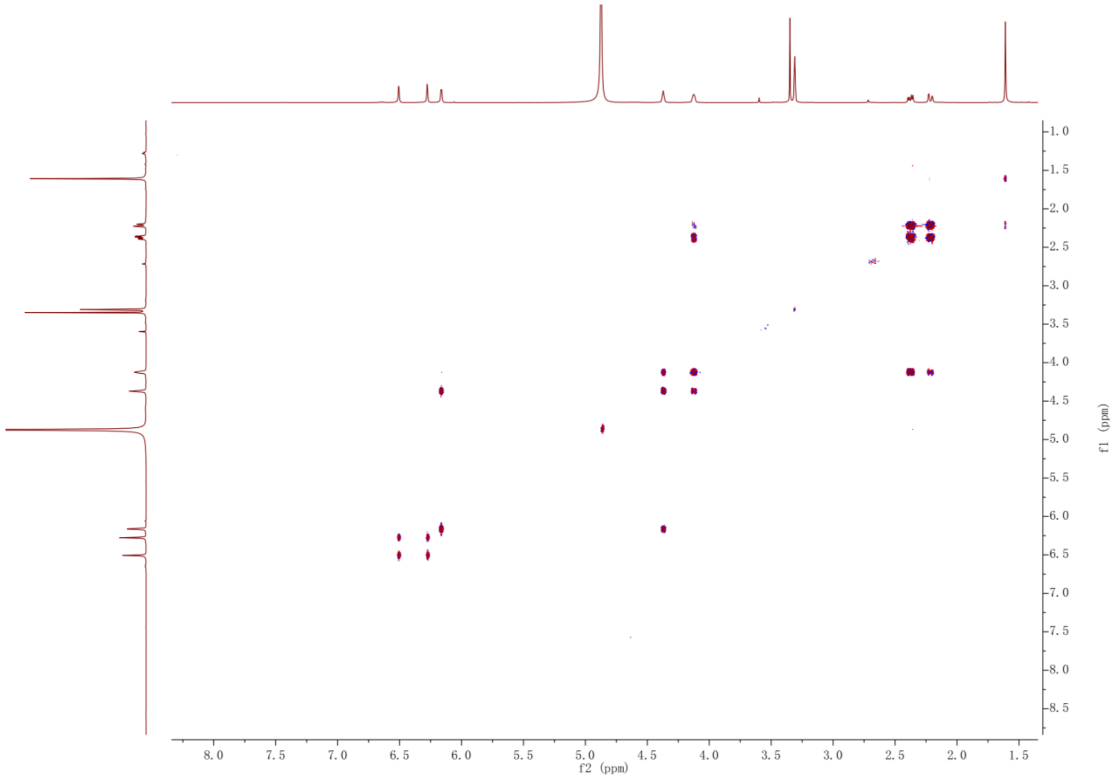

Figure S13. ^1H-^1H COSY spectrum of alternolide B (**2**; 500 MHz, CD_3OD)

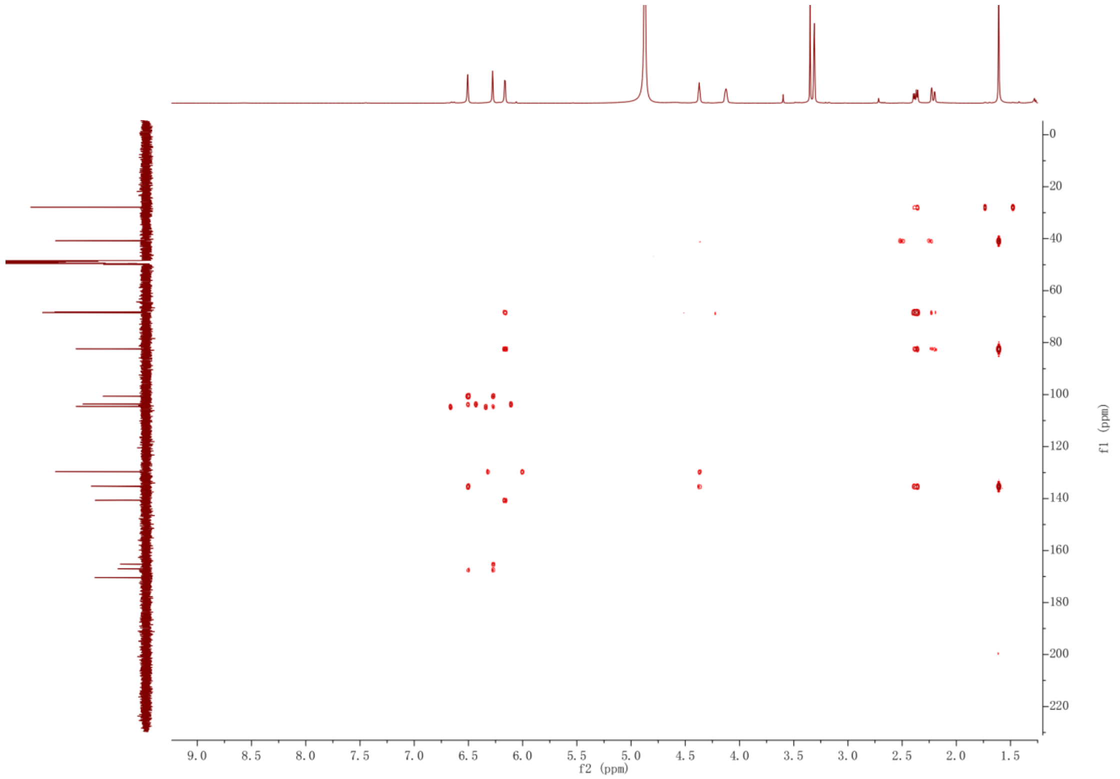

Figure S14. HMBC spectrum of alternolide B (**2**; 500 MHz, CD_3OD)

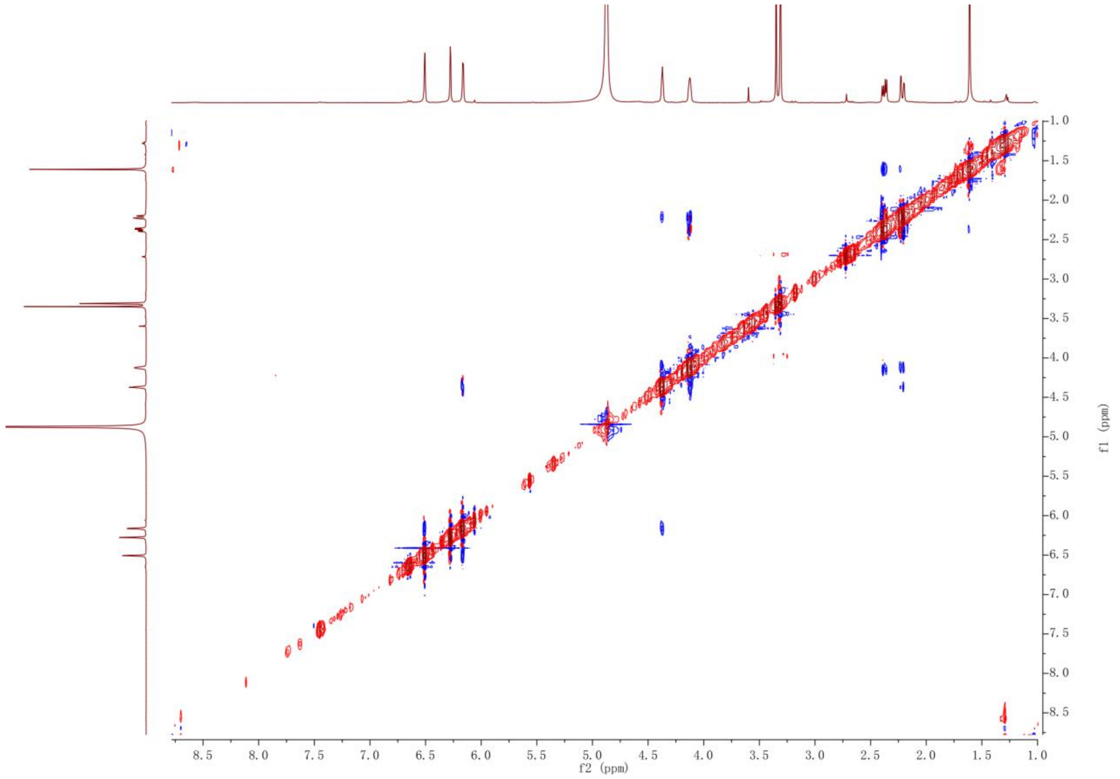

Figure S15. NOESY spectrum of alternolide B (**2**; 500 MHz, CD_3OD)

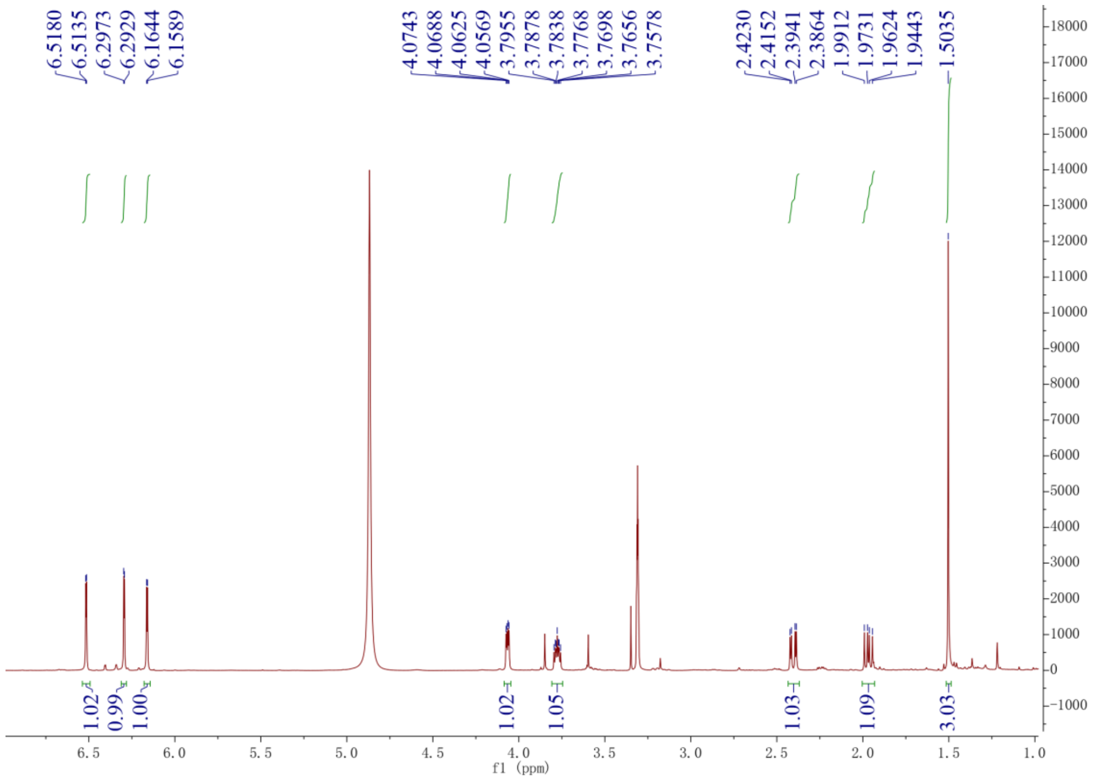

Figure S16. ^{1}H NMR spectrum of alternolide B (**3**; 500 MHz, CD_3OD)

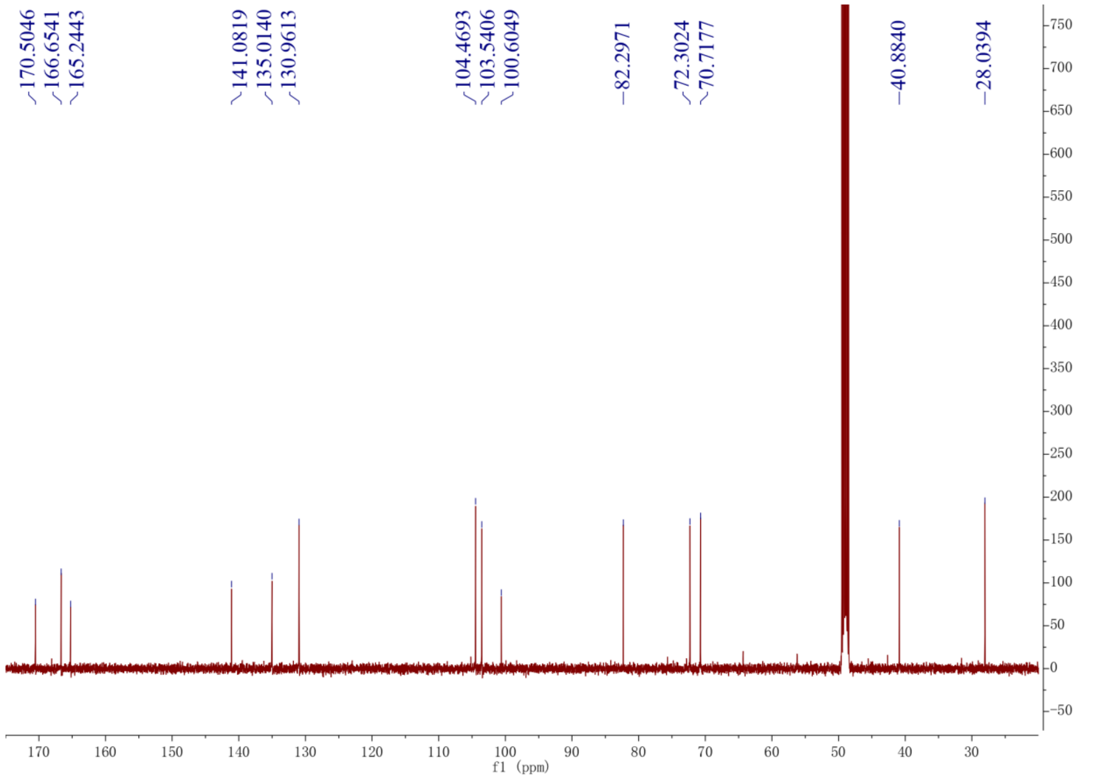

Figure S17. ^{13}C NMR spectrum of alternolide C (**3**; 125 MHz, CD_3OD)

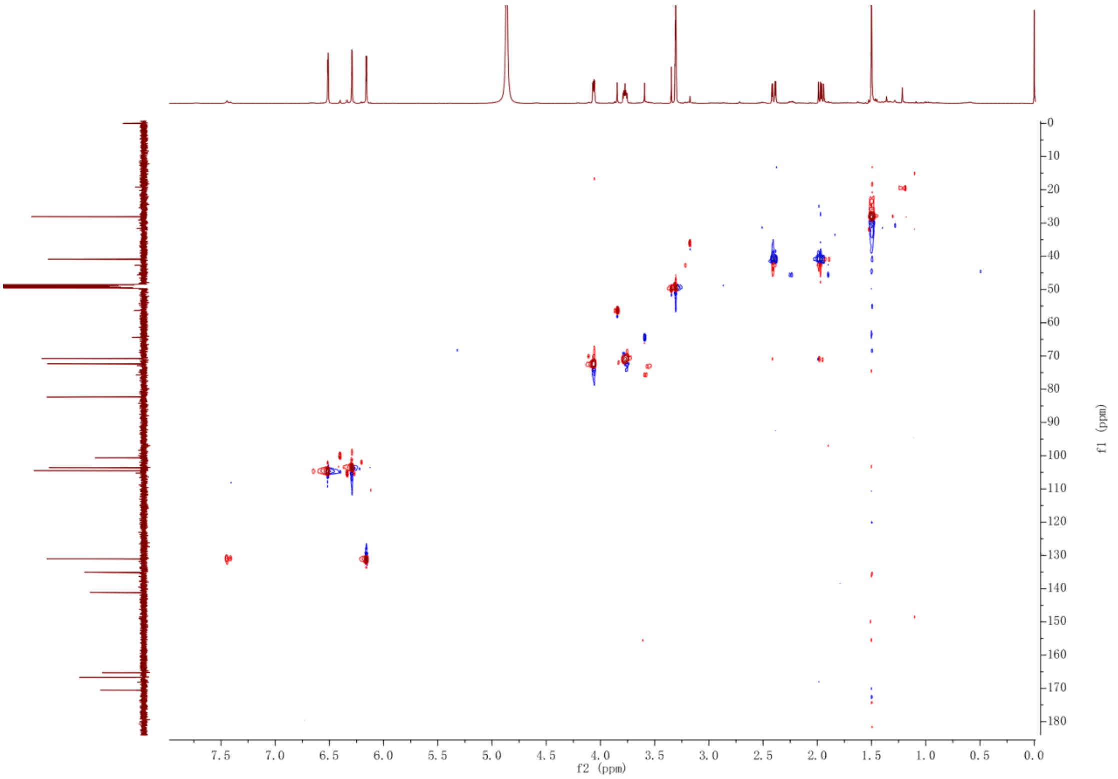

Figure S18. HSQC spectrum of alternolide C (**3**; 500 MHz, CD_3OD)

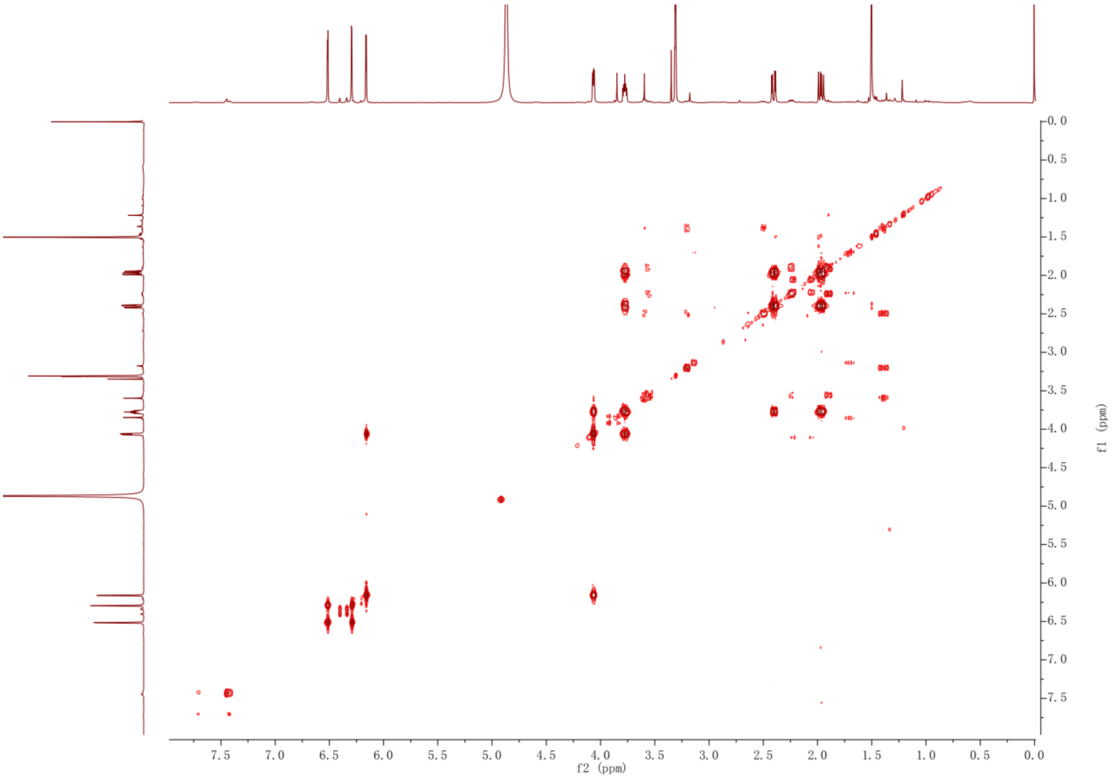

Figure S19. ^{1}H-^{1}H COSY spectrum of alternolide C (**3**; 500 MHz, CD_3OD)

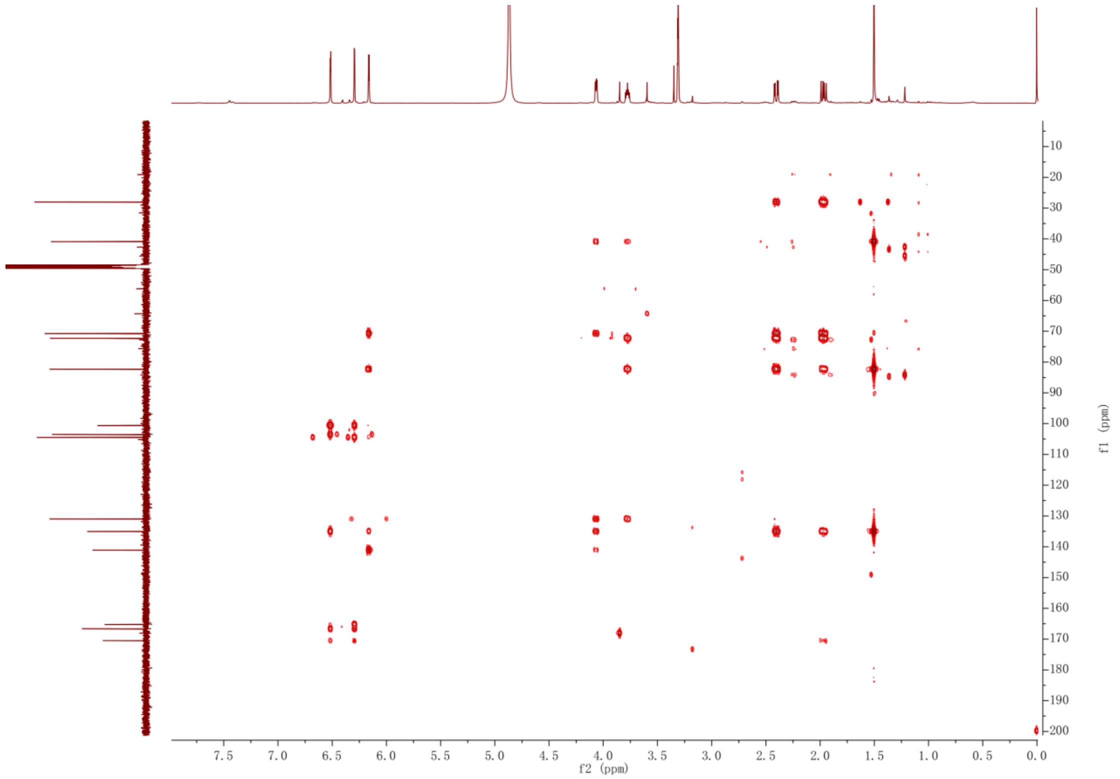

Figure S20. HMBC spectrum of alternolide C (**3**; 500 MHz, CD_3OD)

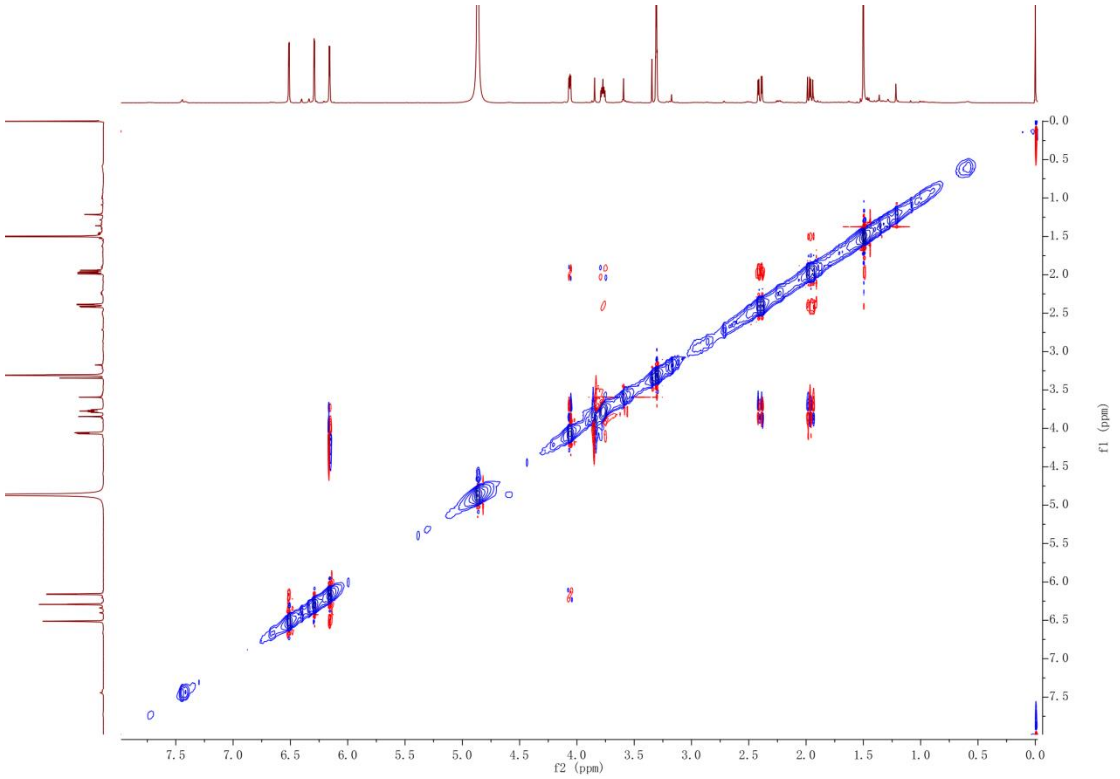

Figure S21. NOESY spectrum of alternolide C (**3**; 500 MHz, CD_3OD)

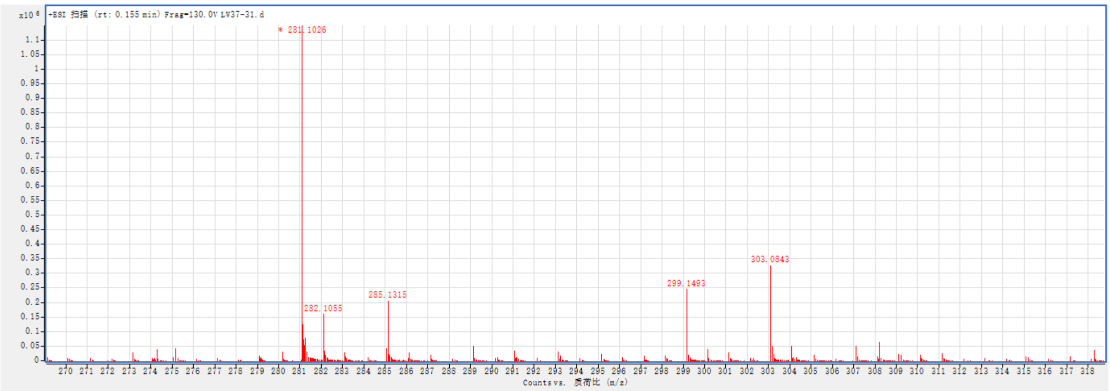

Figure S22. HRESIMS spectrum of alternolide A (**1**)

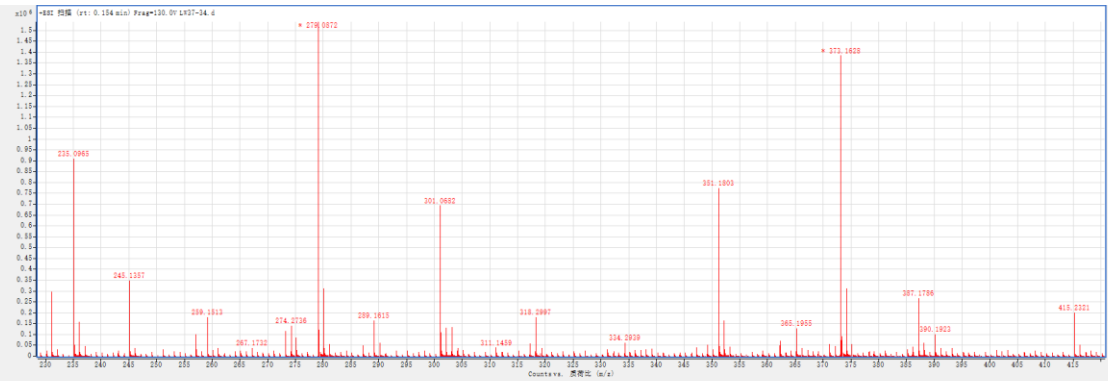

Figure S23. HRESIMS spectrum of alternolide B (**2**)

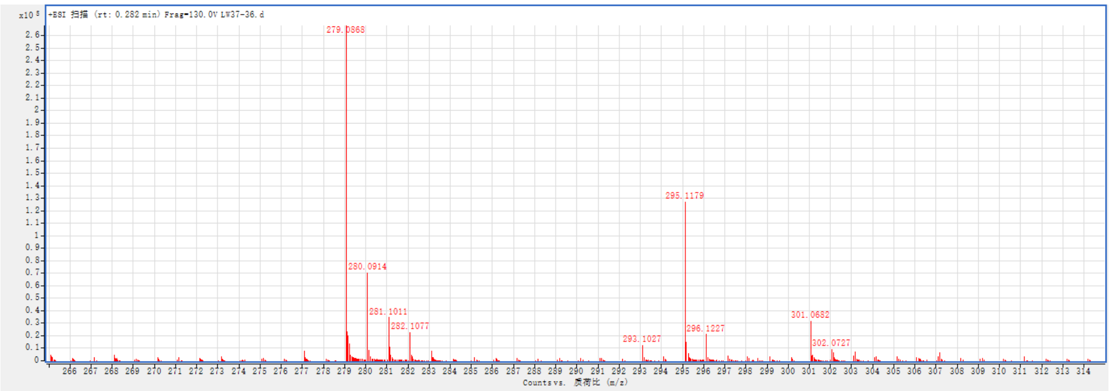

Figure S24. HRESIMS spectrum of alternolide C (**3**)

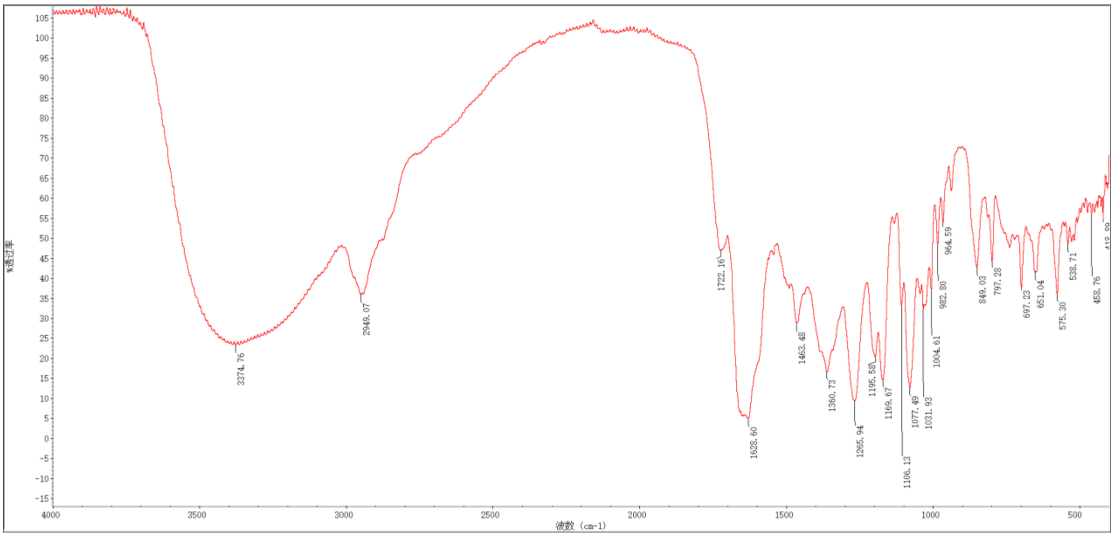

Figure S25. Infrared spectrum of alternolide A (**1**)

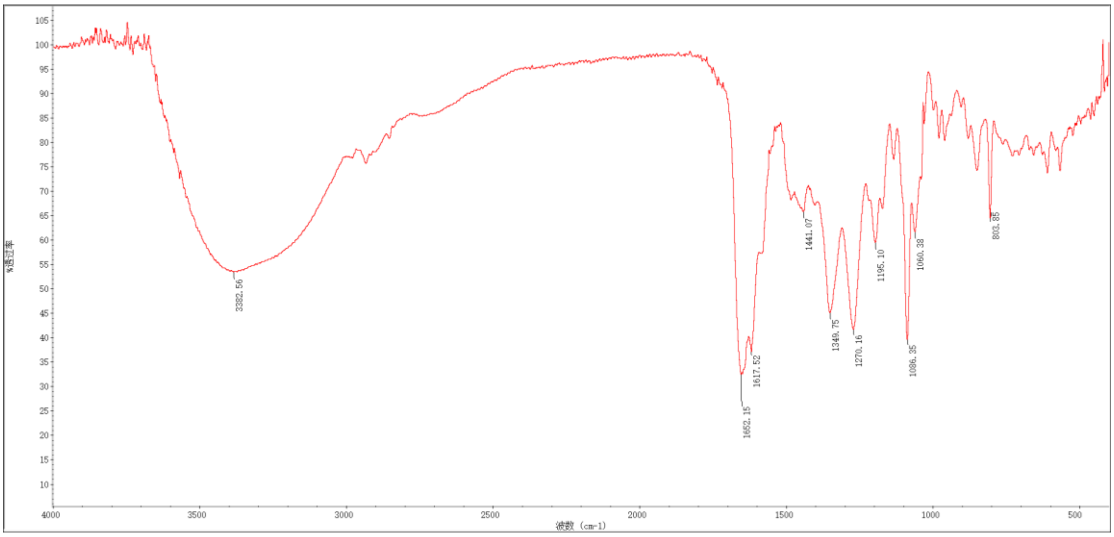

Figure S26. Infrared spectrum of alternolide B (**2**)

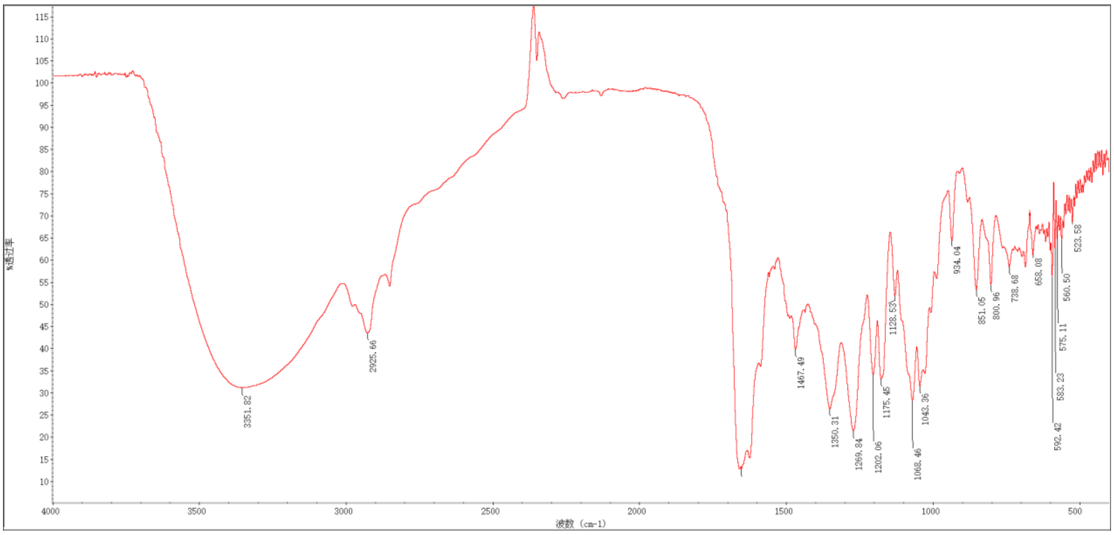

Figure S27. Infrared spectrum of alternolide C (**3**)

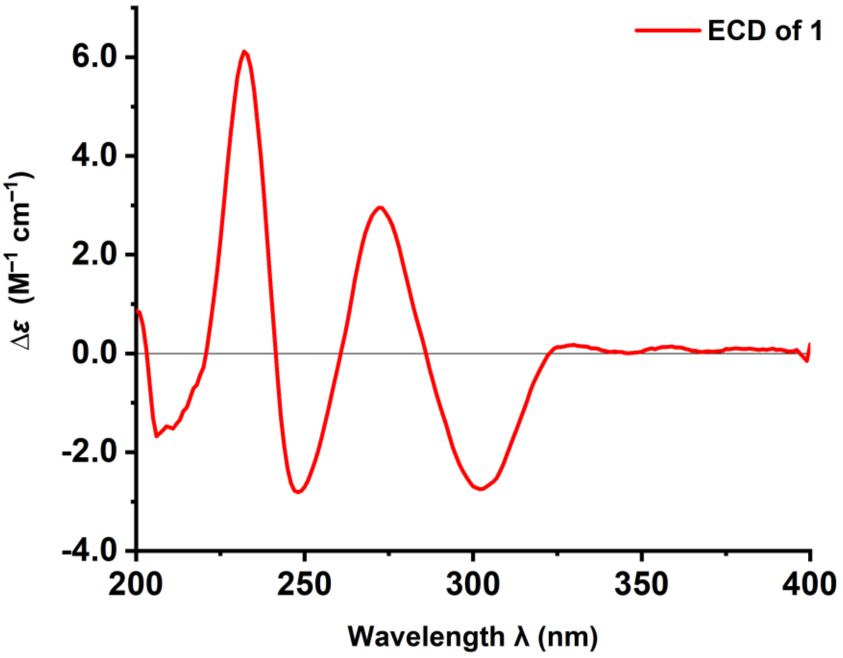

Figure S28. ECD spectrum of alternolide A (**1**)

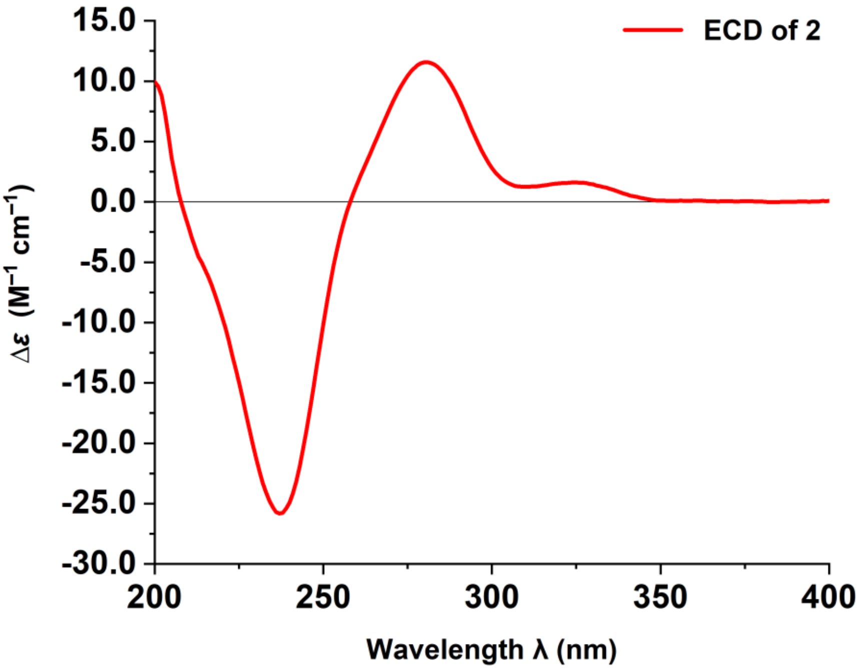

Figure S29. ECD spectrum of alternolide B (**2**)

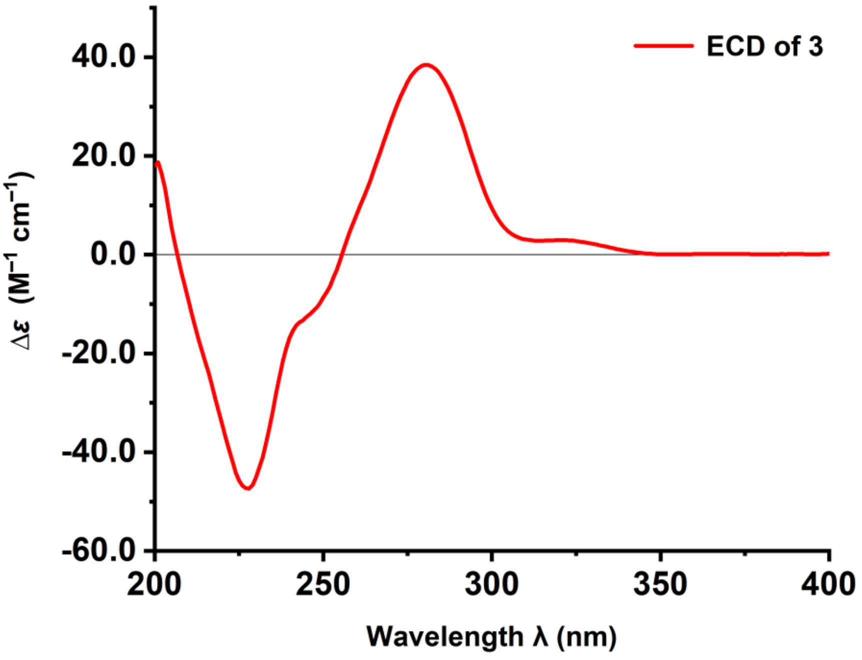

Figure S30. ECD spectrum of alternolide C (**3**)

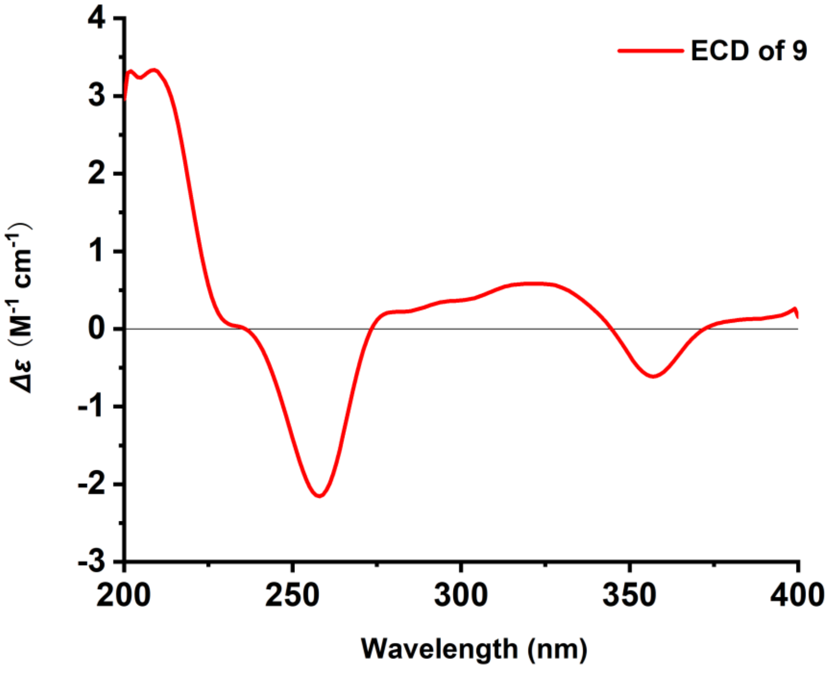

Figure S31. ECD spectrum of 1-deoxyrubralactone (**9**)

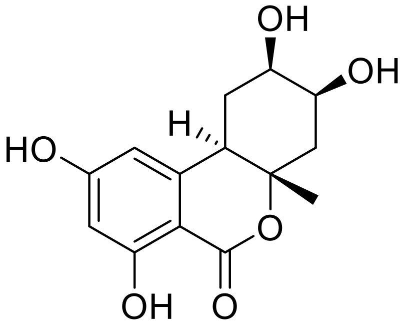

Conformers	Populations
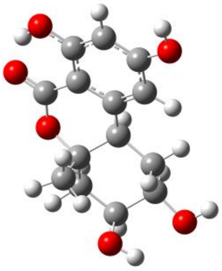	23.15%
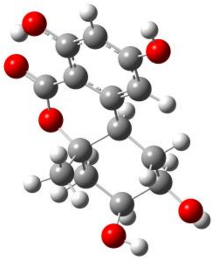	19.31%
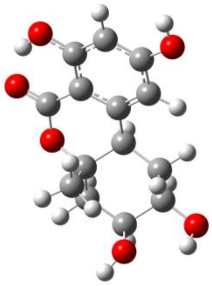	14.47%

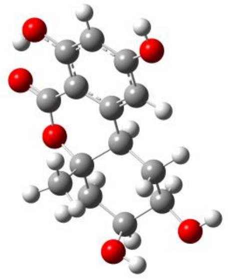	23.48%
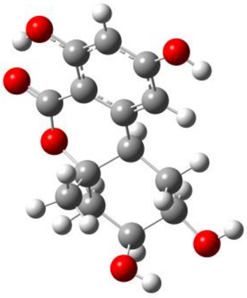	4.17%
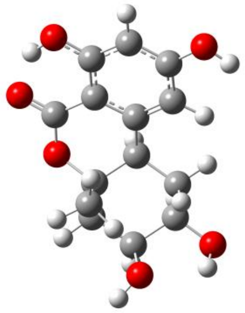	4.99%
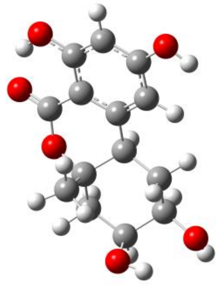	5.50%

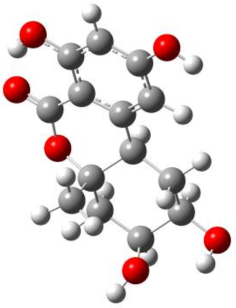	4.93%

Figure S32. ECD conformers of alternolide A (**1**)

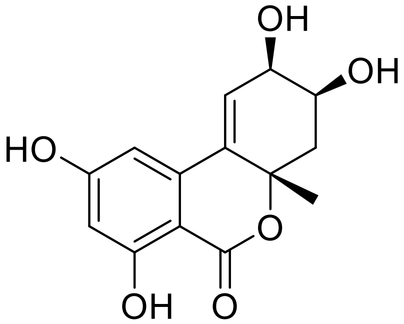

Conformers	Populations
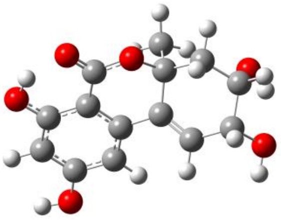	12.38%
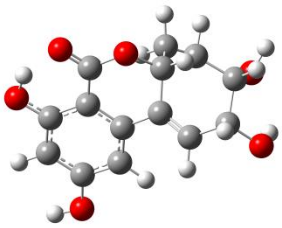	22.21%

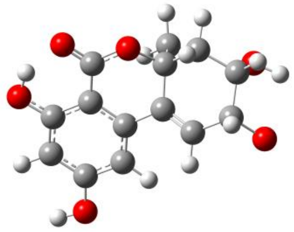	13.70%
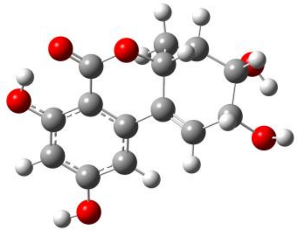	25.30%
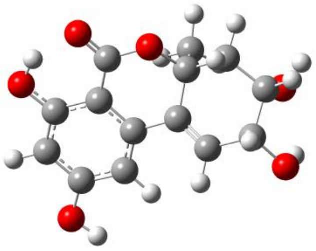	9.38%
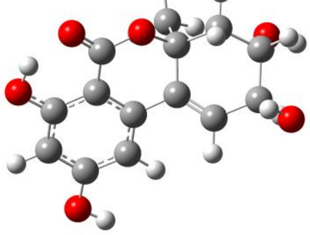	4.14%

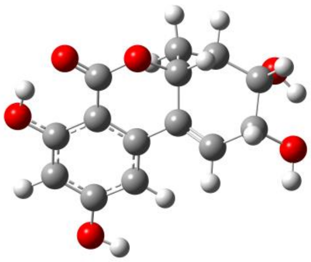	3.08%
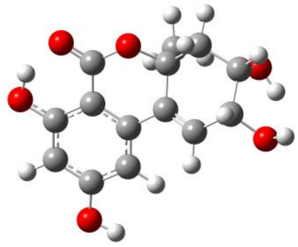	9.81%

Figure S33. ECD conformers of alternolide B (**2**)

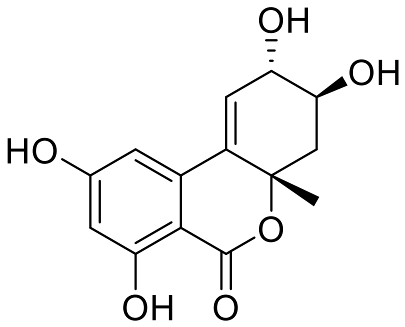	
Conformers	Populations
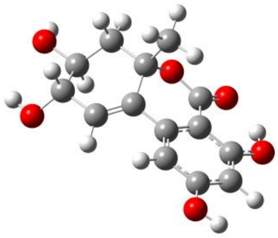	20.44%

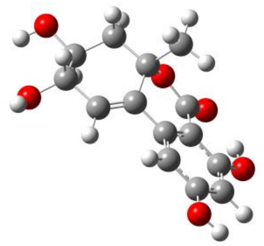	5.85%
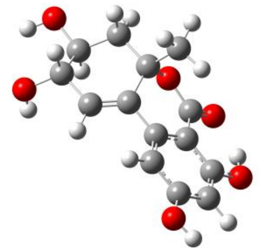	24.00%
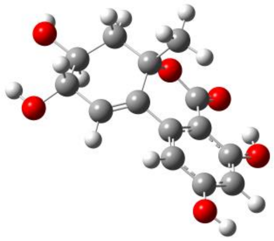	20.11%
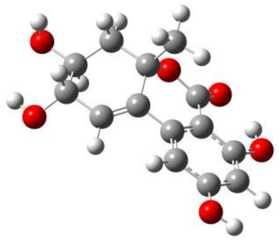	4.73%

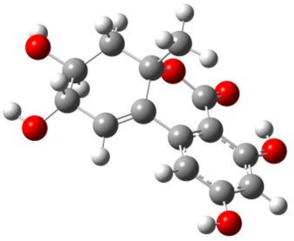	8.02%
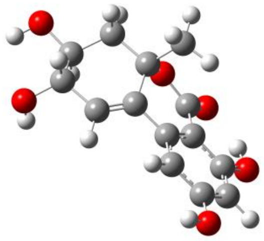	7.48%
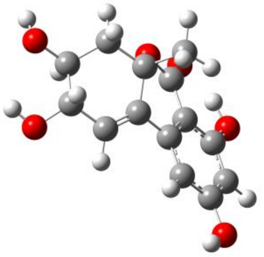	7.90%
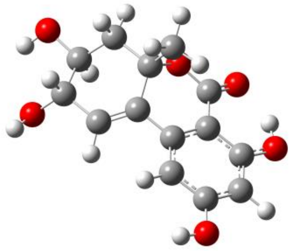	14.6%

Figure S34. ECD conformers of alternolide C (**3**)

<table>
<tr><td colspan="2">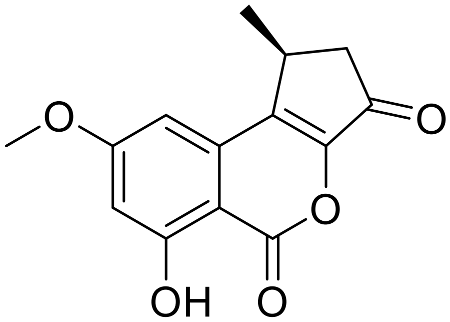</td></tr>
<tr><td>Conformers</td><td>Populations</td></tr>
<tr><td>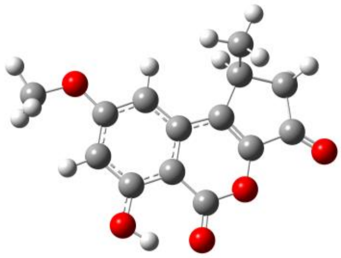</td><td>84.72%</td></tr>
<tr><td>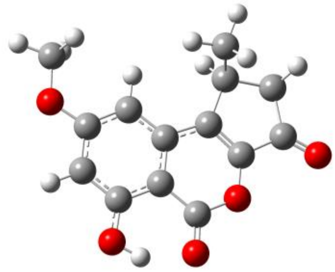</td><td>15.28%</td></tr>
</table>

Figure S35. ECD conformers of 1-deoxyrubralactone (**9**)
